# Differences in ionic currents between canine myocardial and Purkinje cells

**DOI:** 10.1002/phy2.36

**Published:** 2013-08-22

**Authors:** Mario Vassalle, Leonardo Bocchi

**Affiliations:** Department of Physiology and Pharmacology, State University of New YorkDownstate Medical Center, 450 Clarkson Avenue, Brooklyn, NY, 11203

**Keywords:** Cardiac electrophysiology, ionic currents, single ventricular myocardial and cardiac Purkinje cells, whole cell patch clamp method

## Abstract

An electrophysiological analysis of canine single ventricular myocardial (VM) and Purkinje (P) cells was carried out by means of whole cell voltage clamp method. The following results in VM versus P cells were obtained. I_N__a3_ was present, had a threshold negative to the fast activating–inactivating I_N__a1_, its slow inactivation was cut off by I_N__a1_, and contributed to Na^+^ influx at I_N__a1_ threshold. I_N__a1_ was smaller and had a less negative threshold. There was no comparable slowly inactivating I_N__a2_, accounting for the shorter action potential. Slope conductance at resting potential was about double and decreased to a minimum value at the larger and less negative I_K__1_ peak. The negative slope region of I-V relation was smaller during fast ramps and larger during slow ramps than in P cells, occurred in the voltage range of I_K__1_ block by Mg^2+^, was not affected by a lower V_h_ and TTX and was eliminated by Ba^2+^, in contrast to P cells. I_C__a_ was larger, peaked at positive potentials and was eliminated by Ni^2+^. I_to_ was much smaller, began at more positive values, was abolished by less negative V_h_ and by 4-aminopyridine, included a sustained current that 4-aminopyridine decreased but did not eliminate. Steeper ramps increased I_K__1_ peak as well as the fall in outward current during repolarization, consistent with a time-dependent block and unblock of I_K__1_ by polyamines. During repolarization, the positive slope region was consistently present and was similar in amplitude to I_K__1_ peak, whereas it was small or altogether missing in P cells. The total outward current at positive potentials comprised a larger I_K__1_ component whereas it included a larger I_to_ and sustained current in P cells. These and other results provide a better understanding of the mechanisms underlying the action potential of VM and P cells under normal and some abnormal (arrhythmias) conditions.

## Introduction

The different functions of Purkinje (P) and ventricular myocardial (VM) cells are associated with several electrophysiological and mechanical differences (e.g., see Lin and Vassalle 1978; Cordeiro et al. 1998). Thus, the action potential (AP) of canine P fibers is longer (+71%, Lin and Vassalle 1978), their plateau is more negative (e.g., Baláti et al. 1998) and their twitch is shorter (−40%) and smaller (−79%) (Lin and Vassalle 1978) than in ventricular myocardial fibers.

The longer AP of P cells appears related to a greater Na^+^ influx during the plateau through the slowly inactivating sodium current I_Na2_ (Vassalle et al. 2007; Bocchi and Vassalle 2008). Indeed, the Purkinje fiber AP is markedly shortened by tetrodotoxin (TTX; Coraboeuf et al. 1979; Vassalle and Bhattacharyya 1980; Bhattacharyya and Vassalle 1982; Iacono and Vassalle 1990; Baláti et al. 1998) and by local anesthetics (Vassalle and Bhattacharyya 1980; Bhattacharyya and Vassalle 1981), whereas is prolonged by high [Na^+^]_o_ and the Na^+^-channel agonist veratridine (Iacono and Vassalle 1990). In contrast, AP duration of ventricular myocytes is very little affected by TTX (Coraboeuf et al. 1979; Bhattacharyya and Vassalle 1982; Iacono and Vassalle 1990; Baláti et al. 1998), by local anesthetics (Vassalle and Bhattacharyya 1980), by veratridine and high [Na^+^]_o_ (Iacono and Vassalle 1990).

These findings suggest that sodium influx during the action potential may be greater in Purkinje fibers because it also includes I_Na2_, which slowly inactivates at plateau potentials (Vassalle et al. 2007; Bocchi and Vassalle 2008). In addition, in P cells the slowly inactivating sodium current I_Na3_ is activated at potential negative to that of I_Na1_ threshold (Rota and Vassalle 2003). Whether I_Na2_ and I_Na3_ are also present in VM cells or whether Na^+^ currents have identical features in P and VM cells have not been determined.

Furthermore, it is not known whether there are differences in negative slope (NS) and positive slope (PS) regions of the I-V relation between the two tissues. In P cells, I_Na3_ and I_Na2_ are involved in the NS region (Rota and Vassalle 2003), but the role of the block and unblock of inward rectifying I_K1_ channels (Ishihara 1997; Ishihara and Ehara 1998) in the NS and PS regions, respectively, is undefined. Furthermore, whether the mechanisms underlying NS and PS region are similar or differ in P and VM cells is unknown.

There are differences in electrophysiological features of other currents as rabbit P cells express smaller I_K1_, a larger transient outward current I_to_ than VM cells (Cordeiro et al. 1998) and a greater I_to_ sensitivity to TEA (Han et al. 2000). Whether voltage-and time-dependent features of I_K1_, I_to_, sustained current, and I_Ca_ differ in P and VM cells have not been determined.

The general aim of the present experiments was to investigate several ionic currents by means of a whole cell patch clamp method in canine P and VM cells isolated with the same technique to determine their features (e.g., presence or absence, threshold potential, magnitude, time- and voltage-dependent characteristics).

The specific aims included the determination of differences in the following features in P and VM cells: (1) I_K1_ inward rectification and its characteristics; (2) slope conductance over the voltage range of the action potential; (3) presence of I_Na3_ and I_Na2_ and their characteristics; (4) I_Na1_ amplitude; (5) threshold potential for different Na^+^ currents and their voltage- and time-dependent inactivation; (6) contribution of I_Na3_ to peak I_Na1_; (7) presence and magnitude of NS and PS regions and their underlying mechanisms; (8) I_to_ peak and sustained outward current; (9) magnitude and voltage range of the inward component related to I_Ca_; and (10) identification of the various currents by different means including different V_h_, different ramps slopes, and channel blockers.

It was found that the differences in ionic currents between P and VM cells are numerous and substantial and provide insights in the different mechanisms that shape the action potentials, in their modification by some physiological and pharmacological factors and in the mechanisms of induction of some ventricular arrhythmias.

## Material and Methods

Institutional and national guide for the care and use of laboratory animals was followed. The protocols for the experiments were reviewed and approved by the local Animal Care and Use Committee.

The details of the methods have been published (Rota and Vassalle 2003; Vassalle et al. 2007; Bocchi and Vassalle 2008). In brief outline, adult dogs (beagle, *n* = 25) of either sex were euthanized by intravenous injection of sodium pentobarbital (60 mg kg^−1^). Once the respiration had stopped, the hearts were removed and rinsed in physiological saline solution. Purkinje fiber bundles and thin papillary muscles or trabeculae (diameter ≤ 1 mm) were cut from both ventricles and were driven at 60/min for 30 min while being superfused in a tissue bath at 37°C.

The composition of physiological saline solution in mmol L^−1^ was NaCl 140, KCl 5.4, CaCl_2_ 1.8, MgCl_2_ 1, HEPES 5.0, and glucose 5.5. The solution was gassed with 100% O_2_ and adjusted to pH 7.4 with NaOH. The P and VM fibers were then rinsed with Ca-free solution with added 25 mmol L^−1^ taurine, 5 mmol L^−1^ beta-hydroxybutyric acid and 5 mmol L^−1^ Na pyruvate for 5 min in the same tissue bath and washed in a test tube three times with the same Ca-free solution. Ca-free solution contained in mmol L^−1^: NaCl 140, KCl 5.4, KH_2_PO_4_ 1.2, MgCl_2_ 1.5, HEPES 5.0, and glucose 5.5 (pH adjusted to 7.2 with NaOH).

P and VM tissues were separately digested at 37.5°C in Ca-free physiological saline solution to which collagenase (1 mg/mL, type VIII, Sigma, St. Louis, MO), elastase (0.6 mg/mL, type II-A, Sigma), and essentially fat-free bovine serum albumin (2 mg/mL) had been added (“enzyme solution”). The cells were separated from the digested fibers by agitation by means of a mechanical “triturator” (Datyner et al. 1985). The cells were suspended in Kraftbrühe (KB) solution and samples of the cell suspension were perfused with physiological saline solution at 37°C in a chamber located on the stage of an inverted microscope (Nikon Diaphot, Nikon, Tokyo, Japan).

Whole cell patch clamp technique was employed using an Axopatch 1D amplifier. The pipettes were filled with the following solution (in mmol L^−1^): K-aspartate 100, KCl 30, MgCl_2_ 2.0, EGTA 11.0, Na-HEPES 10.0, Na_2_-ATP 2.0, NaGTP 0.1, CaCl_2_ 5.0 (pH 7.2) (resistance of filled pipettes 2–4 MΩ). The free Ca^2+^ in the pipette solution was 110 nmol L^−1^ as calculated using a computer program (WinMAXC 2.40; http://stanford.edu/cpatton/maxc.html). The electrical signals were digitized at 333 kHz 12-bit resolution using A/D converter (Digidata 1200, Axon Instruments, Foster City, CA) and recorded using Clampex software (pCLAMP 8.0, Axon Instruments) and low-pass filtering at 2 kHz.

We elected to study ionic current profiles under physiological conditions (intact intracellular and extracellular ionic concentration and absence of channel blockers). Although this approach does not allow to fully isolate single currents, it preserves ionic balances and electrochemical gradients during the acquisition. Therefore, the currents in P and VM cells were studied in the absence of any channel blocker (such as Ba^2+^, Ni^2+^, tetrodotoxin, 4-Aminopyridine, etc.) to compare and contrast the currents under physiological conditions and to avoid the multiple effects of channel blockers on currents and ionic gradients. Later on, we identified the current under study and their role on different parameters in different ways, including different V_h_, different ramps slopes, and channel blockers.

Successive command steps of the same protocol were applied at intervals of at least 5 sec and different protocols were separated by intervals of 3–5 min to allow the effects of each procedure to fully subside.

The data were analyzed by means pCLAMP program (Axon Instruments Inc.). Steps from different holding potentials (V_h_) were applied to activate voltage- and time-dependent currents and depolarizing and repolarizing ramps with different slopes were used to study the currents under different conditions. On step depolarization from V_h_ −80 mV, I_Na1_ was often cut off at −10 nA by the saturation of the amplifier. As no differences were detected in the results obtained from male and female dog cells, the results were pooled together.

The amplitude of the slowly decaying component of I_Na2_ was measured as the difference between the current at the beginning and the end of the step. The beginning was taken as the value at the intersection between the rapidly inactivating I_Na1_ and the backward extrapolation of I_Na2_, also checked by fitting the slowly inactivating I_Na2_ with a double exponential function.

Unless otherwise specified, the current traces were fitted with two term standard exponential function using the Chebyshev technique with Clampfit software according to equation (1):


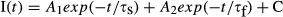
(1)

where A_1_ and A_2_ are the amplitudes, and τs and τf are the time constants and C is the offset constant.

Data were analyzed by mean of the Clampfit (pCLAMP 10.2) and Microsoft Excel programs. The results of tests carried out for each procedure are shown in the tables as means ± SEM (standard error of the mean) together with the number (*n*) of cells studied. Student's paired *t* test between two terms of comparison and one-way ANOVA (analyses of variance) between a data group were applied and a *P* < 0.05 was considered significant and was marked by an asterisk (*) in the tables and in text.

## Results

### I_Na3_ and its relation to I_Na1_

In P cells, I_Na3_ appears at a potential (−57.8 mV) which is negative to I_Na1_ threshold (−52 mV) (Rota and Vassalle 2003). I_Na1_ suppresses the slow inactivation of I_Na3,_ as at its threshold I_Na1_ is not followed by time-dependent current (Rota and Vassalle 2003; Vassalle et al. 2007; Bocchi and Vassalle 2008). Whether I_Na3_ also is present in VM cells or whether its slow inactivation is suppressed by I_Na1_ is not known.

**Figure 1:**
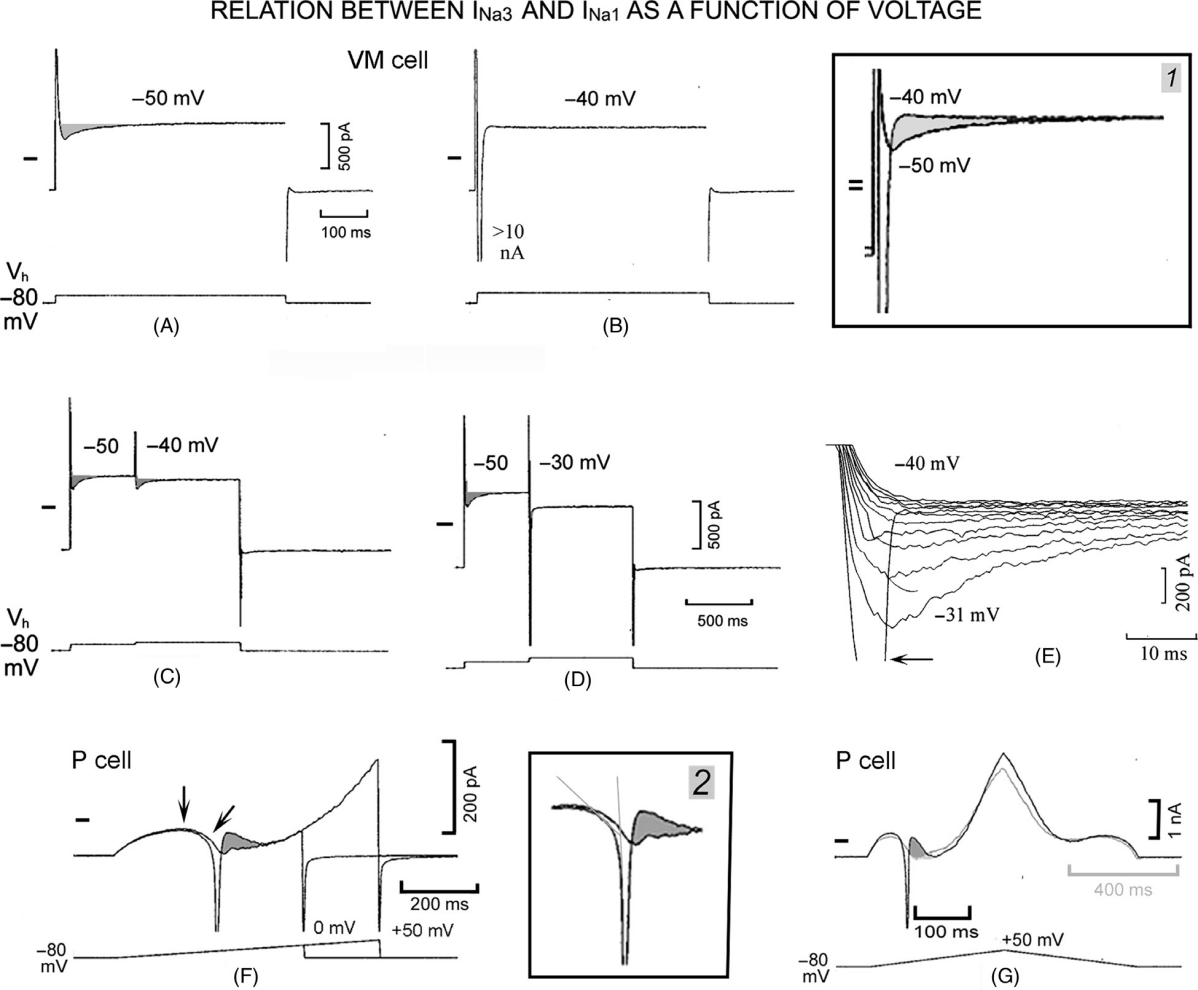
I_Na3_ and its relation to I_Na1_. In a VM cell, a depolarizing step from V_h_ −80 mV to −50 mV (lower trace in A) and to −40 mV (B) elicited the currents shown in the upper traces. The current traces have been superimposed in inset 1 and the shaded area emphasizes the suppression of the slow inactivation of I_Na3_ by I_Na1_. In C, a conditioning step was applied to −50 mV and a test step to −40 mV and in D to −50 and −30 mV, respectively. In E, depolarizing steps were applied from V_h_ −80 to −40 mV and increased by 1 mV to −30 mV: the horizontal arrow points to inactivating I_Na1_. In F, in a P cell, ramps with the same slope and different duration were applied. Downward vertical arrow points to I_K1_ peak and downward oblique arrow to the negative slope region. In inset 2, part of the F traces are shown at greater gain. Dashed lines emphasize the different slopes of inward currents prior to and at beginning of I_Na1_. In F and G, the shaded areas show that at the end of I_Na1_ inactivation the magnitude of the outward current approached that of I_K1_ peak. The short dash in each panel indicates zero current in this and subsequent figures. In G, ramps with different slopes (260 mV sec^−1^_,_ gray trace; 520 mV sec^−1^, black trace) were applied.

In Figure 1A, in a VM cell during the step from V_h_ −80 mV to −50 mV, an inward current appeared that decayed bi-exponentially. In Figure 1B, the step to −40 mV elicited I_Na1_ (partially shown), which (as in P cells) was not followed by a slowly inactivating component. In Figure 1 inset 1, the shaded area emphasizes the fact that I_Na3_ slow inactivation was present at −50 mV and absent at −40 mV.

The absence of I_Na3_ during the step at I_Na1_ threshold potential could be due to either the suppression of I_Na3_ slow inactivation by I_Na1_ (as in P cells) or to the less negative voltage. To clarify this point, a two-step protocol was applied, as a suitable conditioning step may reduce I_Na1_ channel availability just enough to shift its threshold to a less negative value. In Figure 1C, the conditioning step to −50 mV induced I_Na3_ and the test step to −40 mV failed to activate I_Na1_ and induced a smaller I_Na3_. The finding suggests that in Figure 1B I_Na3_ inactivation was not present because it was suppressed by I_Na1_ and not because it could not occur at −40 mV.

In Figure 1D, the −30 mV test step initiated an inward transient (−3037 pA) which was followed by a small shallow tail (−93 pA), suggesting the induction of I_Na2_ with a small slow inactivation component.

In *n* = 10, with the two steps protocol in VM cells during the step from −80 mV to −50 mV I_Na3_ amplitude was −143.2 ± 54.9 pA and during the test step to −40 mV it was −72.4 ± 9.9 pA (not significantly different). During the test step to −30 mV, the inward transient was −3045 ± 576 pA and was followed by a decaying tail of 63.7 ± 14.8 pA. Therefore, in VM cells I_Na3_ was present during the −40 mV test step in the absence of I_Na1_. During the −30 mV test step, I_Na2_ was followed by a small and quickly inactivating component (see below). Similarly, in P cells (*n* = 10) I_Na3_ could be activated at the I_Na1_ threshold if the activation of I_Na1_ was prevented by the conditioning step. One difference with the VM cells was that in P cells the slowly inactivating I_Na2_ was much larger (+673.1%*; see below).

I_Na3_ was studied in VM and P cells by applying single steps (Fig. 1A) from V_h_ −80 mV (Table 1). With respect to P cells, in VM cells during depolarizing steps I_Na3_ was consistently present, had a less negative threshold (*), and similar amplitude as well as time constants of inactivation.

**Table 1:** I_Na3_ in P and VM cells and its changes with lower V_h_

V_h_ (mV)	Param	VM cells	P cells
−80	Th (mV)	−46.7 ± 1.1	−53.3 ± 1.9*
	I_Na3_ (pA)	−168 ± 52 (18/18)	−217 ± 102 (13/18)
	τ_f_ (msec)	15.3 ± 2.9	10.5 ± 2.8
	τ_s_ (msec)	82.7 ± 11.9	55.9 ± 12.9
−70	Th (mV)	−43.3 ± 1.3	−52.5 ± 1.8*
	I_Na3_ (pA)	−189 ± 66 (15/16)	−190 ± 63 (11/16)
−60	Th (mV)	−38.5 ± 1.0	−46.6 ± 1.2*
	I_Na3_ (pA)	−120 ± 29 (12/15)	−235 ± 71 (14/15
−50	Th (mV)	−33.6 ± 1.5	−35.6 ± 2.0
	I_Na3_ (pA)	−179 ± 76 (11/16)	−155 ± 42 (14/16)
−40	Th (mV)	−21.0 ± 1.0	−27.1 ± 1.8*
	I_Na3_ (pA)	−29.9 ± 18.3 (3/10)	−84 ± 35 (6/9)

V_h_ (mV), holding potential in mV; Param, parameters measured; VM cells, data from ventricular myocardial cells; P cells, data from Purkinje cells; Th (mV), threshold potential in mV of I_Na3_; I_Na3_ (pA), amplitude in pA of I_Na3_ measured as the difference between its peak and the end of the step; τ_f_ (msec) and τ_s_ (msec), fast and slow time constants, respectively, of I_Na3_ inactivation; Numbers in parenthesis (e.g., 18/18), number of cells in which I_Na3_ was present over the total number of cells studied; *statistically significant difference between P and VM cells data.

The finding that the −50 mV conditioning step prevented the appearance of I_Na1_ but not of I_Na3_ during the −40 mV test step suggests that I_Na3_ might be less sensitive to voltage-dependent inactivation than I_Na1_. This was tested by applying depolarizing steps from gradually less negative V_h_. As shown in Table 1, in P and VM cells with gradually less negative V_h_, the amplitude of I_Na3_ decreased very little until V_h_ was −40 mV and the threshold remained less negative (*) in VM cells. At all V_h_, in both P and VM cells the inactivation of I_Na3_ was slow in the absence of I_Na1_. Even with V_h_ −40 mV, I_Na3_ inactivated with τ_f_ 13.1 ± 7.5 msec and τ_s_ 41.3 ± 18.5 msec in VM cells and with τ_f_ 7.5 ± 1.4 msec and τ_s_ 82.1 ± 22.6 msec in P cells.

### I_Na3_ voltage-dependent increase and sudden suppression of its slow inactivation by I_Na1_

Gradually increasing depolarizing steps might lead to a progressive increase in I_Na3_. In Figure 1E, in a VM cell with depolarizing steps increasing by 1 mV between the I_Na3_ and I_Na1_ thresholds, I_Na3_ magnitude increased progressively and inactivated relatively more quickly up to −29 mV. At −30 mV (I_Na1_ threshold), the inactivation of I_Na1_ (arrow) suppressed I_Na3_ slow decay. Similar results with steps increasing at intervals of 1 mV were obtained in VM cells (*n* = 4) and in P cells (*n* = 16).

The above results suggest that during depolarizing ramps I_Na3_ might precede I_Na1_, as the continuous decline in voltage would initiate and increase I_Na3_ at potentials negative to the I_Na1_ threshold. Furthermore, the current at the end of I_Na1_ inactivation would be expected to be more outward than in its absence due to the suppression of I_Na3_ slow inactivation. In some cells, applying ramps of different duration with a borderline slope (150 mV sec^−1^) for I_Na1_ activation led to a nonuniform induction of I_Na1_, so that the events in the presence and absence of I_Na1_ could be compared in the same cell as shown in Figure 1F.

In a P cell, during the ramps, the outward current gradually increased before peaking (I_K1_ peak, vertical arrow). The slope of the ramps being borderline for I_Na1_ activation, during the shorter ramp, I_K1_ peak was followed by a negative slope (NS) region (oblique arrow). No I_Na1_ was present and the current during the NS region (I_NS_) was followed by a reincreasing outward current. During the longer ramp, I_NS_ more quickly turned inward and its steeper slope merged into that of the activating I_Na1_, as emphasized by the gray lines in Figure 1 inset 2.

In Figure 1F and inset 2, the shaded area shows that the current at the end of I_Na1_ inactivation was more outward than in the I_Na1_ absence, as expected from the suppression of the slow inactivation of I_Na3_ by I_Na1_. The inactivation of I_Na1_ was still followed by NS region with smaller amplitude and less steep slope, consistent with the slow inactivation of I_Na2_ (see below). The patterns illustrated in Figure 1F were present in *n* = 3, the consistent presence of I_Na1_ with its inactivation approaching I_K1_ peak in *n* = 4 and the absence of I_Na1_ with the consistent presence of I_NS_ in *n* = 19.

In another approach, ramps with different slopes (Fig. 1G) were applied, as in several instances in P cells no I_Na1_ was initiated during slower ramps. In Figure 1G, the 260 mV sec^−1^ ramp (lighter trace) did not activate I_Na1_, whereas the superimposed 520 mV sec^−1^ ramp (darker trace) did. During the slower ramp, I_K1_ peak was followed by I_NS_ but not by I_Na1_. Instead, with the steeper ramp, the end of I_Na1_ inactivation approached the I_K1_ peak and was followed by I_NS_.

The asymmetry between slower activation and faster inactivation of the overall Na^+^ current (Fig. 1 F and G) was a consistent finding that might be expected from I_Na3_ preceding I_Na1_ and its slow inactivation being cut off by it.

In VM cells, I_Na1_ was less frequently absent with slower ramps. With 260 mV sec^−1^ ramps, I_Na1_ was absent in 9/17 P cells and in 3/17 VM cells whereas with the 520 mV sec^−1^ ramp, I_Na1_ was absent only in 3/17 P cells and in none of 17 VM cells (the NS region being present with or without I_Na1_).

Therefore, in VM cells I_Na3_: (1) was present with a similar magnitude and rate constants of inactivation; (2) had a threshold less negative than in P cells and negative to that of I_Na1_; (3) increased progressively at potentials between its threshold and that of I_Na1_; (4) its slow inactivation was consistently eliminated by I_Na1_; (5) could appear and inactivate slowly at voltages less negative than I_Na1_ threshold if I_Na1_ activation was prevented by a conditioning step; (6) contributed to the beginning of I_NS_ during depolarizing ramps, and (7) was less sensitive than I_Na1_ to voltage- and time-dependent inactivation.

### Currents during larger depolarizing steps in Purkinje and myocardial cells

**Figure 2:**
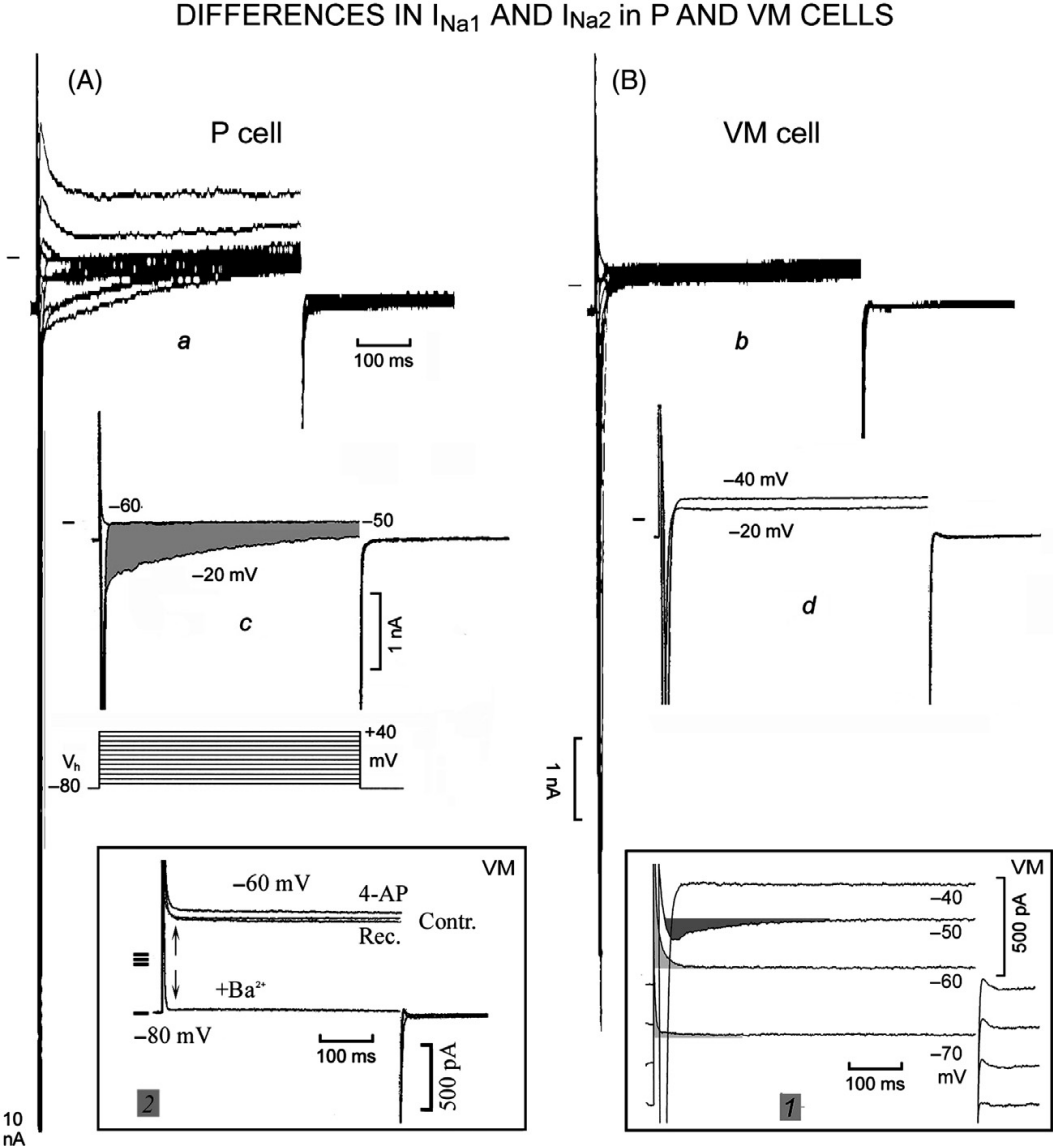
Larger range of inward and outward currents in P than in VM cells. The protocol is shown at the bottom of c. In the P cell, the currents flowing during the steps from V_h_ −80 to −60, −50, and −20 mV are shown in c, where the shaded area emphasizes the slow decay of I_Na2_. In the VM cell, the currents recorded with steps from V_h_ −80 to −40 and −20 mV are shown in d. In inset 1, the current traces were displaced by 200 pA for a better visualization. The light-shaded areas emphasize the time-dependent component of I_K1_ and the dark-shaded area I_Na3_. In inset 2, the current traces during the −60 mV step are shown in control (Contr.) in the presence of 4-aminopyridine (4-AP), of 4-AP plus Ba^2+^ (+Ba^2+^), and during recovery (Rec.). The upward arrow indicates the initial decline in the outward current to a steady value and the downward arrow its suppression by Ba^2+^.

Ionic currents in P and VM cells were investigated also by applying 500 msec depolarizing steps from V_h_ −80 mV to +40 mV in increments of 10 mV (Fig. 2, protocol in c).

In Figure 2, in the P cell (A) the range of inward and outward currents was larger than in the VM cell (Fig. 2B). In P cell, I_Na1_ was truncated by the saturation of the amplifier at −10,000 pA whereas in VM cell the largest I_Na1_ was −8428 pA. In the P cell (Fig. 2c), the −50 mV step initiated I_Na1_ whose inactivation was followed by a steady current that overlapped the current trace at −60 mV (where no I_Na1_ was present; see also Vassalle et al. 2007; Bocchi and Vassalle 2008), thus suggesting no loss of voltage control. The slowly inactivating I_Na2_ appeared at −40 mV and reached its largest value during −20 mV step (shaded area in Fig. 2c). Typically, I_Na2_ was still decreasing by the end of the 500 msec step (Vassalle et al. 2007; Bocchi and Vassalle 2008).

In the VM cell, the threshold for I_Na1_ activation was less negative (−40 mV, Fig. 2d) than in the P cell (−50 mV). During the depolarizing −20 mV step, the inactivation of I_Na1_ was not followed by a decaying I_Na2_, in contrast to the P cell. The comparison of Figure 2a and b indicates that in VM cell the large and slowly decaying I_Na2_ was absent at other potentials as well. The sustained current at the end of −50 mV step (measured as the difference from the holding current I_h_) was larger in the VM cell than in the P cell. In the VM cell, the sustained current at −40 mV (I_Na1_ threshold) was similar to that at −50 mV (not shown). In both cells, the sustained current at −20 mV was less outward than at −50 mV (P cell) and at −40 mV (VM cell), as expected from the onset of I_NS_. With more positive steps, the outward currents were far larger in P cell than in VM cell (cf. Fig. 2a and b).

In Table 2, with V_h_ −80 mV, in VM cells I_Na1_ threshold was less negative in P cells (*), I_Na1_ was smaller (*) in VM cells, as it was truncated in all P cells, but only in 13/18 VM cells. In both VM and P cells, I_Na1_ inactivated exponentially with τ ∼1.5 msec. In VM cells, I_Na1_ was smaller (*) than in P cells also with V_h_ −70 mV when I_Na1_ was less often truncated.

**Table 2:** I_Na1_ in P and VM cells and its changes with lower V_h_

V_h_ (mV)	Param	VM cells	P cells
−80	Th (mV)	−36.7 ± 1.1	−48.3 ± 1.5*
	I_Na1_ (pA)	−8857 ± 461 (18/18)	−10, 000 ± 0.0* (18/18)
	τ (msec)	1.3 ± 0.2	1.6 ± 0.2
−70	Th (mV)	−33.7 ± 1.2	−43.1 ± 1.8*
	I_Na1_ (pA)	−8551 ± 445 (16/16)	−9622 ± 263.0* (16/16)
	τ (msec)	1.5 ± 0.2	1.6 ± 0.2
−60	Th (mV)	−30.7 ± 1.5	−34.7 ± 1.7
	I_Na1_ (pA)	−6167 ± 959 (15/15)	−7984 ± 844 (15/15)
	τ (msec)	1.4 ± 0.2	1.6 ± 0.2
−50	Th (mV)	−23.3 ± 1.4	−26.0 ± 1.3
	I_Na1_ (pA)	−2153 ± 618 (11/16)	−3404 ± 820 (14/16)
	τ (msec)	2.4 ± 0.7	1.6 ± 0.1
−40	Th (mV)	−13.3 ± 3.3	−18.75 ± 1.3
	I_Na1_ (pA)	−58.1 ± 33 (3/10)	−642 ± 325 (5/8)
	τ (msec)	3.7 ± 0.6	2.9 ± 0.4

I_Na1_ (pA), amplitude of I_Na1_ from its beginning to its peak in pA; τ (msec), time constant of the exponential inactivation in ms of I_Na1_; VM cells, data from ventricular myocardial cells; P cells, data from Purkinje cells; Th (mV), threshold potential in mV of I_Na1_; Numbers in parenthesis (e.g., 18/18), number of cells in which I_Na1_ was present over the total number of cells studied; *statistically significant difference between P and VM cells data.

In Table 3, at −20 mV in VM cells an inactivating I_Na2_ tail was present in 6/18 cells, was small and decayed quickly. In contrast, in P cells the slowly inactivating I_Na2_ was much larger (*) and inactivated more slowly (*). With gradually less negative V_h_, I_Na1_ (Table 2) and I_Na2_ (Table 3) gradually decreased.

**Table 3:** I_Na2_ in P and inward tail in VM cells and their changes with lower V_h_

V_h_ (mV)	Param	VM cells	P cells
−80	Peak (mV)	−20.0 ± 0.0	−21.2 ± 0.8
	I_Na2_ slow (pA)	−110.2 ± 39.3 (6/18)	−1212 ± 208* (15/18)
	τ_f_ (msec)	4.0 ± 1.8	8.1 ± 1.0*
	τ_s_ (msec)	60.8 ± 22.1	233.9 ± 24.7*
−70	Peak (mV)	−20.0 ± 0.0	−20.6 ± 0.6
	I_Na2_ slow (pA)	−66.1 ± 25.8 (9/16)	−1985 ± 263* (15/16)
−60	Peak (mV)	−20.0 ± 0.0	−22.1 ± 1.1
	I_Na2_ slow (pA)	−46.1 ± 20.0 (5/15)	−854 ± 140* (13/15)
−50	Peak (mV)	−13.3 ± 2.1	−15.3 ± 1.6
	I_Na2_ slow (pA)	−35.7 ± 18.4 (5/16)	−382 ± 195 (9/16)

Peak (mV), voltage at which the largest slowly inactivating I_Na2_ in P cells or in VM cell was measured; I_Na2_ slow (pA), amplitude of slowly inactivating I_Na2_ in pA, measured from its beginning to the end of the step; VM cells, data from ventricular myocardial cells; P cells, data from Purkinje cells; τ_f_ (msec) and τ_s_ (msec), fast and slow time constants, respectively, of I_Na2_ inactivation; Numbers in parenthesis (e.g., 6/18), number of cells in which I_Na2_ was present over the total number of cells studied; *statistically significant difference between P and VM cells data.

The slope conductance was measured by superimposing small hyperpolarizing pulses on the parent steps in VM cells (*n* = 9). At the I_Na1_ threshold, after the I_Na1_ inactivation, the slope conductance was minimal and did not vary with time, as shown in P cells by Bocchi and Vassalle (2008). This finding also is consistent with no loss of voltage control.

### Time-dependent decay of I_K1_ on depolarization in Purkinje and myocardial cells

In Figure 2, in the same VM cell, the currents during the −70, −60, −50, and −40 mV steps are shown in inset 1. At the beginning of the −70 and −60 mV steps, the outward current quickly declined (light-shaded areas) to a steady value. At −50 mV, I_Na3_ appeared and declined slowly (dark-shaded area), and at −40 mV I_Na1_ quickly activated and inactivated to a steady value.

One possible explanation for the initial decline of the outward current at −60 and −70 mV might be a noninstantaneous block of I_K1_ by polyamines during the depolarizing steps (Ishihara 1997; Ishihara and Ehara 1998), a block which is eliminated by Ba^2+^ (Ishihara and Ehara 1998). In Figure 2 inset 2, the traces during the step to −60 mV were recorded in control (Contr.), in the presence of 4-aminopyridine (4-AP), of Ba^2+^ (+Ba^2+^), and during recovery (Rec.). The upward arrow indicates the initial decline in the outward current to a steady value, a decline that was little affected by 4-AP. Instead, Ba^2+^ suppressed both the initial decay and the steady current during the step (downward arrow).

In Table 4, with V_h_ −80 mV, at the voltages indicated in VM cells the outward current decreased more (*) in VM cells than in P cells. The time constant of the exponential decline was similar (∼6 msec).

**Table 4:** I_K1_ time-dependent decay during depolarizing steps from different V_h_

V_h_ (mV)	Param	VM cells	P cells
−80	Measured at (mV)	−58.8 ± 0.7	−59.6 ± 6.4
	I_K1_ decay (pA)	317 ± 29 (18/18)	237 ± 24* (18/18)
	τ (msec)	6.3 ± 0.9	5.9 ± 1.2
−70	Measured at (mV)	−55.6 ± 1.2	−59.3 ± 0.6*
	I_K1_ decay (pA)	226 ± 3.7 (16/16)	62.9 ± 16.5* (11/16)
−60	Measured at (mV)	−50.0 ± 0.0	−50.0 ± 0.0
	I_K1_ decay (pA)	78.4 ± 26.2 (6/15)	21.1 ± 18.2 (2/15)
−50	Measured at (mV)	−40.0 ± 0.0	−40.0 ± 0.0
	I_K1_ decay (pA)	24.1 ± 15.6 (4/16)	12.8 ± 8.8 (2/16)

Measured at (mV), voltage in mV at which the decay of I_K1_ was measured; I_K1_ decay (pA), amplitude of I_K1_ time-dependent decay at beginning of step; τ (msec), time constant of I_K1_ exponential decay; V_h_ (mV), holding potential in mV; VM cells, data from ventricular myocardial cells; P cells, data from Purkinje cells; Numbers in parenthesis (e.g., 18/18), number of cells in which I_K1_ decay was present over the total number of cells studied; *statistically significant difference between P and VM cells data.

If the decline of the current during the step is indeed due to a time-dependent block of I_K1_ on depolarization, then decreasing V_h_ should reduce the declining current, as increasing degrees of I_K1_ block would occur during the less negative V_h_, prior to the depolarizing step. To test this point, V_h_ was reduced in 10 mV increment to −40 mV. As shown in Table 4, in both P and VM cells with gradually less negative V_h_, the initial decay of the current became gradually smaller and less frequent, and disappeared altogether with V_h_ −40 mV. These findings indicate a time- and voltage-dependent block of I_K1_, which was significantly larger in VM cells at V_h_ −70 and −60 mV.

### Contribution of I_Na3_ to Na^+^ inflow due to I_Na1_

As I_Na3_ occurs also at the I_Na1_ threshold, at that potential I_Na3_ would be expected to precede I_Na1_ and therefore contribute to the peak Na^+^ current. To verify such a possibility, the current traces at the beginning of depolarizing steps were displayed at suitably greater time base.

**Figure 3:**
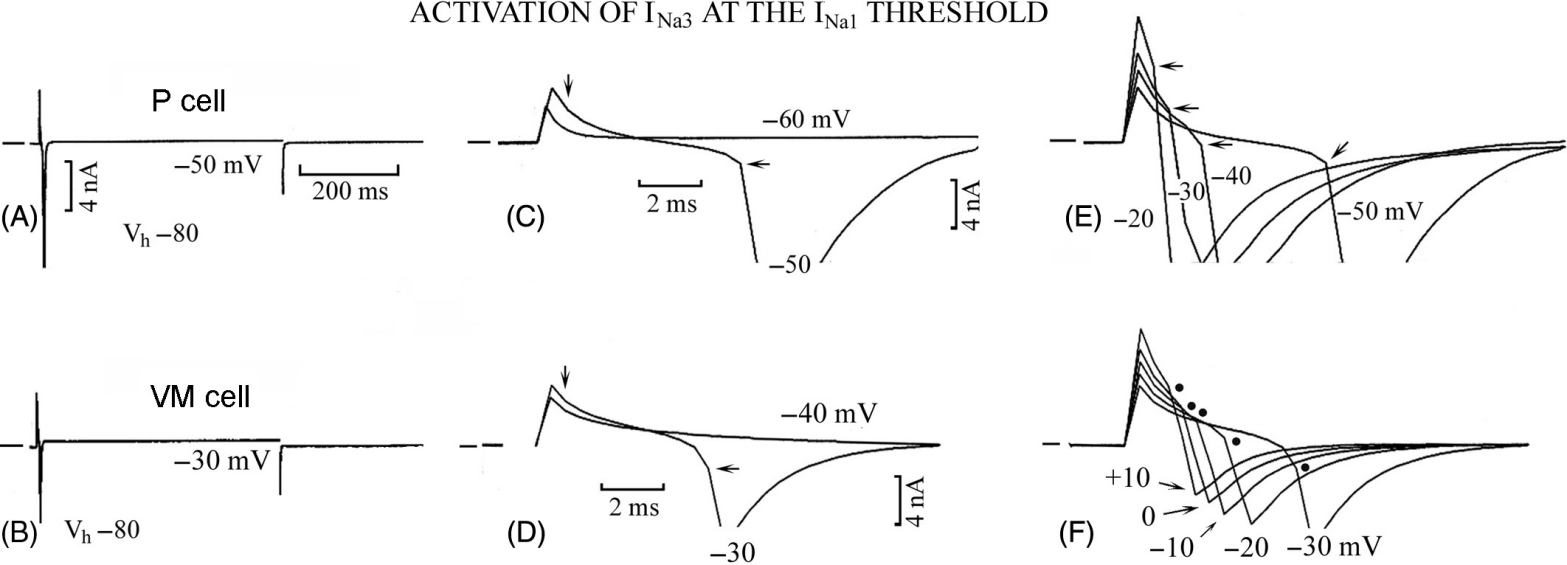
The activation of I_Na3_ precedes that of I_Na1_ at the latter's threshold. In A (P cell) and B (VM cell), the traces are shown at the usual time base whereas in C, D, E, and F the traces are shown at the greater time base indicated. In C and D, the downward vertical arrows point to the beginning of I_Na3_ and the leftward horizontal arrows point to the beginning of I_Na1_. The traces recorded at the beginning of the −50, −40, −30, and −20 mV steps have been superimposed in E (P cell) and those at the beginning of the steps to −30, −20, −10, 0, and +10 mV have been superimposed in F (VM cell). The arrows in E and the dots in F indicate the beginning of I_Na1_. The numbers next to the traces indicate the voltage of the respective steps.

In Figure 3, in a P cell (A) and in a VM cell (B), at the usual time base only I_Na1_ was visible. However, when the traces were displayed at much greater time base, a slower inward component (comprised between the downward and horizontal arrows) preceded I_Na1_ both in P (Fig. 3C) and in VM cell (Fig. 3D). In Figure 3C (P cell), the trace recorded at −60 mV show the initial decay of the outward current to a steady value (time-dependent block of I_K1_). Instead, the trace recorded at −50 mV departed from the exponential decay (vertical arrow), crossed in an inward direction the −60 mV trace (as expected from the activation of I_Na3_). After a delay, it was followed by the fast initiation of I_Na1_ (sudden beginning of the steeper slope, horizontal arrow). Similar events occurred in the VM cell (Fig. 3D).

In Figure 3E (P cell), the current traces at the beginning of −50, −40, −30, and −20 mV steps were superimposed and show the progressively earlier onset of I_Na1_ with larger depolarizing steps until no distinct I_Na3_ component was apparent. In Figure 3F, similar events occurred in the VM cell in that larger depolarizing steps elicited an earlier I_Na1_ with the eventual disappearance of I_Na3_.

In *n* = 18, in VM cells, I_Na3_ (measured between the departure of the trace from exponential decay and the sudden onset of I_Na1_) had a magnitude of −2677 ± 201 pA and a duration of 2.2 ± 0.3 msec; I_Na1_ (measured from the sudden increase in steepness to its peak) had a threshold of –36.6 ± 1.1 mV) and an amplitude of −8857 ± 461 pA. In P cells, I_Na3_ had a amplitude of −2330 ± 235* pA and a duration of 2.6 ± 0.8 msec; I_Na1_ had a more negative threshold (−48.3 ± 1.4* mV) and an amplitude >−10,000 ± 0.0* pA.

Thus, I_Na3_ was a substantial fraction of the inward current flowing at the threshold for I_Na1_. With steps to less negative values, I_Na3_ consistently decreased and I_Na1_ was activated sooner.

### Differences in I-V relation of the sustained current in Purkinje and myocardial cells

To investigate the quasi-steady state I-V relations in VM and P cells, the sustained current at the end of 500 msec depolarizing steps from V_h_ −80 mV to +40 mV was measured as the difference from I_h_. The same procedure was applied with less negative V_h_ to determine how the I-V relation would be affected in P versus VM cells.

**Figure 4:**
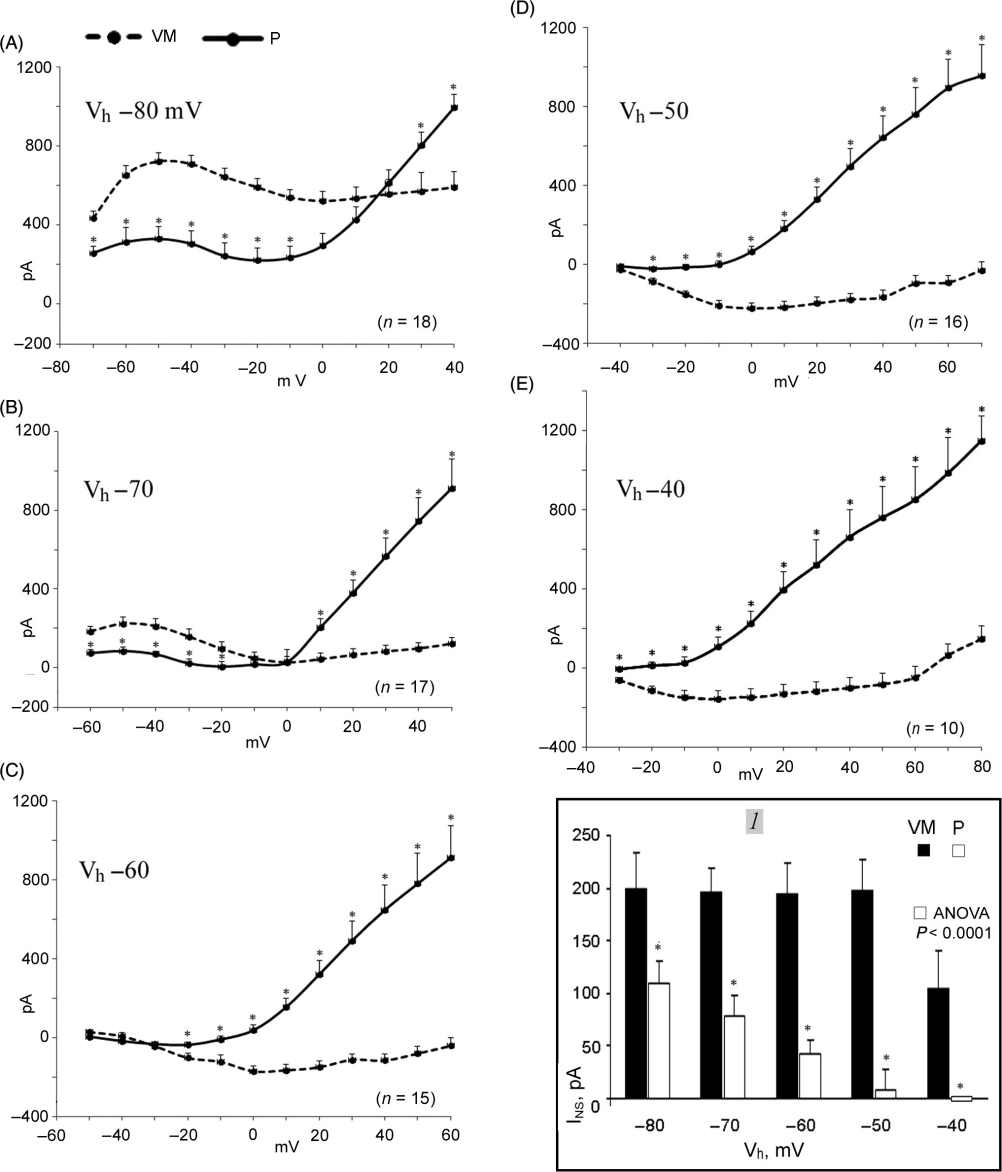
The I-V relation as a function of V_h_ in P and VM cells. The number of P and VM cells studied is indicated in parenthesis in each panel. The ordinates show the magnitude of the sustained current in pA at the end of 500 ms depolarizing steps applied from the V_h_ indicated in each panel to the voltage in mV indicated on the abscissae. The VM cells mean data are connected by dashed line and those of P cells by continuous line. The vertical bars indicate the standard error of the mean. In inset 1, the mean values of I_NS_ are the difference between the most outward sustained current and the subsequent least outward sustained (or the largest inward) current in pA at voltage indicated on abscissa. The asterisks (*) indicate a statistical difference between the data in VM and P cells. The difference between the values in P cells at the various V_h_ was statistically significant (ANOVA *P* < 0.0001).

In Figure 4, in A, with V_h_ −80 mV, in VM cells the sustained current was more outward at negative potentials and less outward at positive potential than in P cells. In both types of cells, past I_K1_ peak, the NS region was present, but in VM cells I_NS_ was larger and peaked at a less negative value (∼0 mV), whereas in P cells the smaller I_NS_ peaked at ∼−20 mV. The outward current began to increase at +10 mV in VM cells and at −10 mV in P cells, suggesting a different I_to_ threshold.

I_NS_ was differently affected by lower V_h_ in P and VM cells. With V_h_ −70 (Fig. 4B), overall the sustained current was much less outward in both VM and P cells, as expected from the inward rectification of I_K1_ channel at less negative V_h_. However, I_NS_ was still large in VM cells whereas it was diminished in P cells, suggesting that in P cells the decrease in I_NS_ might be related to a partial inactivation of I_Na2_.

This interpretation is supported by the findings with still lower V_h_. In VM cells, the current became inward and I_NS_ persisted unaltered up to V_h_ −50 mV (Fig. 4B–D). Only with V_h_ −40 mV did I_NS_ decrease (Fig. 4E) as apparently the channel contributing to I_NS_ was partially blocked prior to the depolarizing step. Instead, in P cells I_NS_ markedly decreased with V_h_ −60 to disappear altogether with V_h_ −40 mV. In Figure 4 inset 1, the graph shows the difference between the outward current peak prior to the NS region (corresponding to I_K1_ peak) and the smallest current value of I-V relation prior to the reincrease in outward current (a measure of I_NS_ peak). The graph shows how differently I_NS_ amplitude varied in VM and P cells as a function of V_h_, the decrease of I_NS_ in P cells being statistically significant (ANOVA < 0.0001).

The increase in outward current with the larger depolarizing steps was much greater in P than in VM cells (helped in this by the inward shift of the current in VM cells), and it was little affected by less negative V_h_. At each V_h_, the outward current in P cells increased past ∼−20 mV, as expected for I_to_. As the protocol applied at different V_h_ was the same, with the gradually less negative V_h_ the depolarizing steps attained gradually more positive values. Hence, the sustained current with the largest depolarizations increased to similar values in spite of the decreasing V_h_.

These results raise the possibility that in P cells Na^+^ currents might mainly contribute to I_NS_ (with this protocol, I_Na2_) whereas the voltage-dependent block of I_K1_ channel may predominantly determine I_NS_ in VM cells.

### I_Na2_ and the I_Ca_ component in Purkinje and myocardial cells

**Figure 5:**
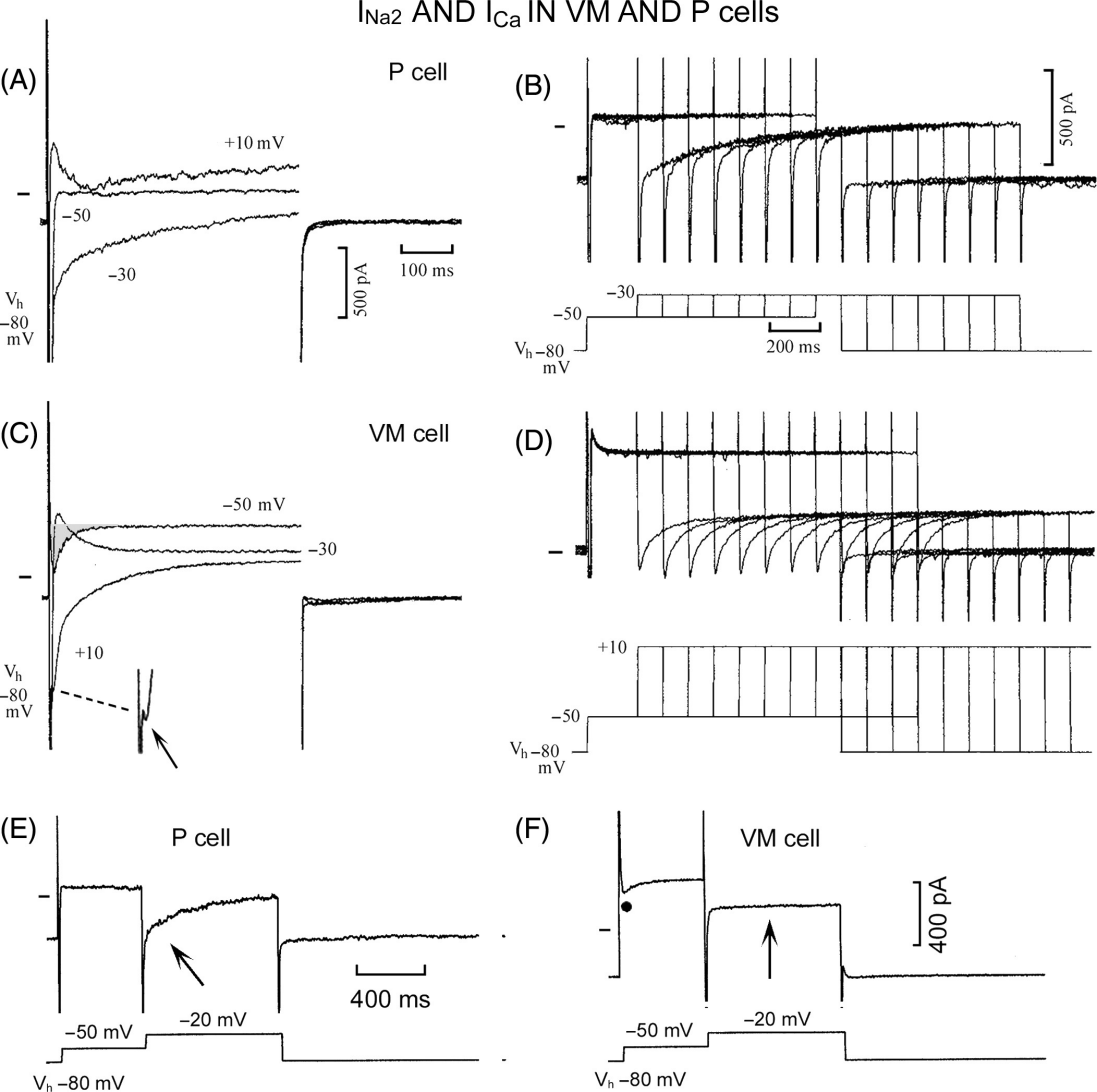
Different amplitude, voltage range, kinetics, and time-dependent inactivation of I_Na2_ in P cells and of I_Ca_ in VM cells. Steps were applied from V_h_ −80 to −50, −30 mV +10 mV in the P cell (A) and in the VM cell (C) where the shaded area emphasizes I_Na3_ and the small arrow points to the beginning of a large I_Ca_. In the P cell (B), progressively longer conditioning steps to −50 mV were followed by test steps to −30 mV. In the VM cell (D), conditioning steps to −50 mV were followed by test steps to +10 mV. In E, the oblique upward arrow points to inactivating I_Na2_ during the test step to −20 mV in the P cell. In F, the filled circle labels I_Na3_ and the vertical upward arrow points to the absence of comparable slowly inactivating I_Na2_ at −20 mV in the VM cell.

As at plateau voltages the slowly inactivating I_Na2_ prevails in P cells and presumably I_Ca_ prevails in VM cells, the different amplitude, voltage range, voltage- and time-dependent inactivation of I_Na2_ in P cells and of the I_Ca_ component in VM cells were investigated as shown in Figure 5.

In the P cell (Fig. 5A), a step from V_h_ −80 to −50 mV elicited I_Na1_ which was not followed by time-dependent currents, as usual. During the step to −30 mV, I_Na1_ was followed by the slowly decaying I_Na2_ (τ_s_ 297 msec). During the +10 mV step, a small inward component was superimposed on a small outward current. In the VM cell (Fig. 5C), the step from V_h_ −80 to −50 mV elicited I_Na3_ (shaded area). During the step to −30 mV, I_Na1_ was followed by a small outward component, but not by decaying I_Na2_. During the +10 mV step, I_Na1_ was followed by a large I_Ca_ component (−1699 pA) which inactivated with a τ_s_ of 106 msec and whose beginning during I_Na1_ inactivation is indicated by the arrow in the magnified trace.

In the P cell (Fig. 5B), after progressively longer conditioning steps at −50 mV, the test steps to −30 mV elicited a gradually smaller inactivating I_Na2_ (with the last test step, −47%) (see Bocchi and Vassalle 2008). In the VM cell (Fig. 5D), the conditioning step was the same, but (as there was no I_Na2_ at −30 mV) test steps were applied to the voltage where I_Ca_ component was largest (+10 mV, protocol in Fig. 5D). The test step elicited an inward current, whose amplitude was not decreased by progressively longer conditioning steps (with the last test step, +3.7%). In the VM cell, I_Ca_ decayed more quickly than I_Na2_ did in the P cell. The findings point to a different voltage range, kinetics, and voltage- and time-dependent inactivation of I_Na2_ in P cells and of I_Ca_ in VM cells.

In order to separate I_Na1_ from I_Na2_, a double step protocol was applied from V_h_ −80 mV to −50 and to −20 mV in a P cell (Fig. 5E) and in a VM cell (Fig. 5F). In the P cell, at −50 mV I_Na1_ was followed by a steady current, and at −20 mV I_Na2_ activated rapidly and decayed slowly (oblique arrow). In the VM cell, at −50 I_Na3_ was present as usual at that voltage (filled circle; see Fig. 1) and at −20 mV an inward transient was followed by a faint and brief tail at a potential where the slowly decaying I_Na2_ was large in the P cell.

With V_h_ −80 mV, in VM cells at +20 mV the I_Ca_ component was −436.7 ± 105.2 pA (17/17 cells) with τ_f_ 19.1 ± 8.4 msec and τ_s_ 112 ± 15.4 msec, whereas in P cells at +18.8 mV the I_Ca_ component was −96.8 ± 44.5* pA (present in 6/17 cells) with τ_f_ 11.4 ± 6.3 msec and τ_s_ 157.6 ± 61.2* msec.

### I-V relation during slow depolarizing and repolarizing ramps in myocardial and Purkinje cells

Because in VM cells the sustained current at the end of depolarizing steps was larger at potentials negative to I_K1_ peak and smaller at positive potentials than in P cells (Fig. 4), the steady state I-V relation was studied during slowly depolarizing and repolarizing ramps in the two tissues.

**Figure 6:**
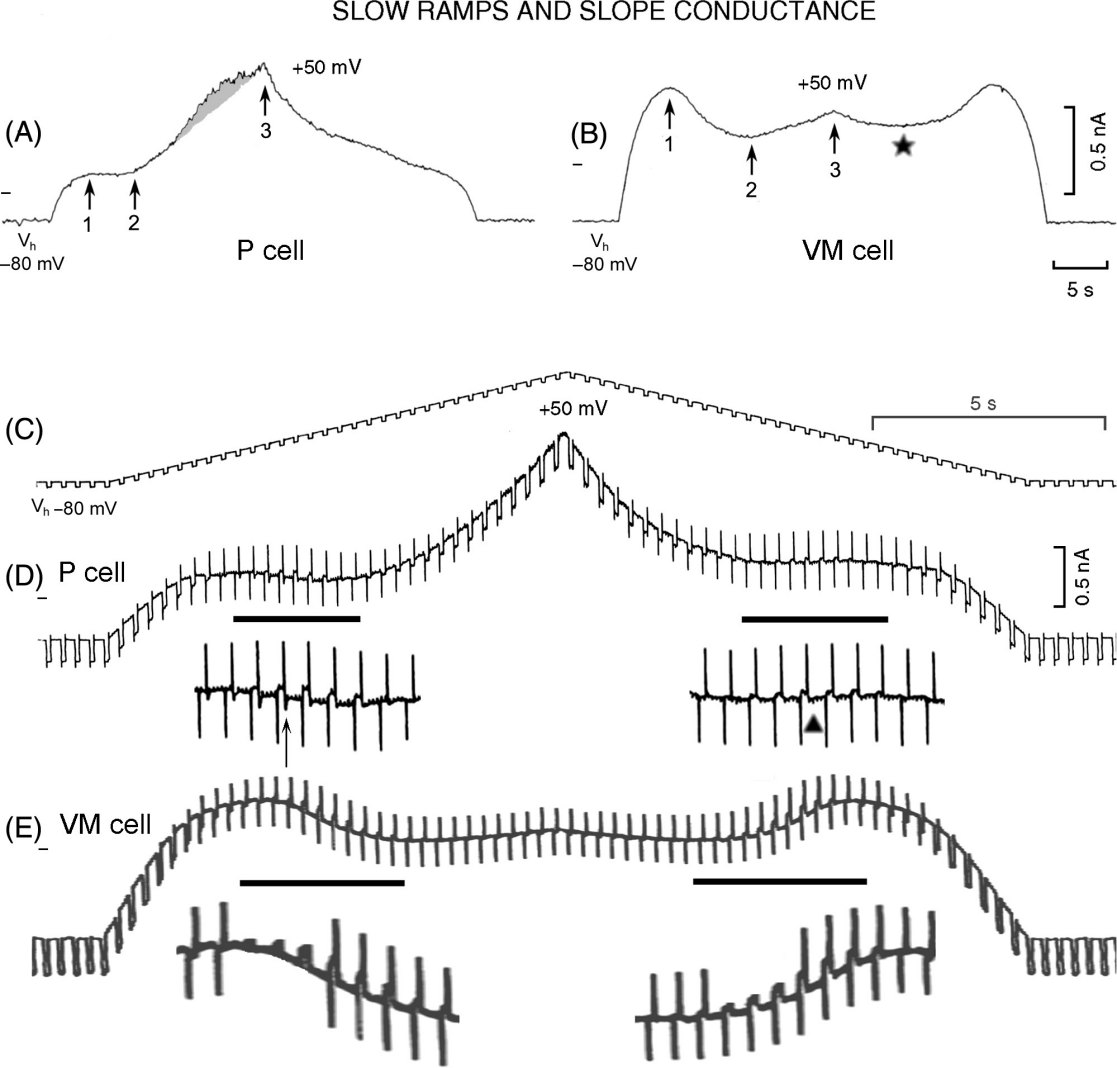
I-V relation and changes in slope conductance during slow ramps in P and VM cells. A 6.5 mV sec^−1^ depolarizing and repolarizing ramp was applied to a P cell (A) and to a VM cell (B). Point 1 indicates the I_K1_ peak, point 2 the beginning of increasing outward current, and point 3 the current at ramp peak. In A, the shaded area indicates the enhancement of the outward current (“the bulge”). In B, the asterisk indicates the transition between the decreasing outward current and the beginning of I_PS_. Hyperpolarizing voltage pulses (amplitude 7 mV, duration 200 msec, rate 90 min^−1^; C) were superimposed on the parent ramp to measure slope conductance. In D (P cell) and E (VM cell) (same heart but different from that for A and B), the horizontal lines indicate the sections of current records shown at higher gain underneath. In D, the upward vertical arrow points to an inward transient after the reversed pulse current and the triangle points to its absence. In the enlarged sections of VM trace, some of the capacity spikes have been deleted for a better visualization of the increase and reversal of pulse current in the NS and PS regions.

In Figure 6A, in the P cell during 6.5 mV sec^−1^ depolarizing ramp, the outward current increased gradually less to stop increasing altogether between point 1 (−58 mV, I_K1_ peak) and point 2 (−30.7 mV). On further depolarization between points 2 and 3, the outward current increased markedly and before the ramp peak, underwent an enhancement (shaded area, the “bulge”; Du and Vassalle 1999). During the repolarizing ramp, the outward current initially decreased more rapidly, but 16 sec after the ramp peak it was similar to 16 sec before (at I_K1_ peak) (292 and 275 pA, respectively).

In Figure 6B, in the VM cell, the outward current also increased gradually less as a function of depolarization, but at point 1 (−48 mV, I_K1_ peak) the outward current was 179% larger than in the P cell. Also, a distinct NS region began at point 1 and was followed (change in slope) by an I_Ca_ component that peaked at +2 mV (point 2). Between points 2 and 3, the current reincreased in an outward direction, but it was much smaller than in the P cell. Furthermore, there was no enhancement of the outward current (no “bulge”) and at the ramp peak (point 3) the current was less outward than at point 1, in sharp contrast with the P cell.

During the repolarizing ramp, the outward current decreased much less than in the P cell and it was less inward (star) than at point 2. The outward current reincreased in the positive slope (PS) region to a peak value (786 pA) similar to that of I_K1_ peak (769 pA). Past the peak of I_PS_, the outward current underwent a progressively quicker decrease as a function of repolarization.

In Table 5, with respect to P cells, in VM cells I_K1_ was larger (*) and peaked at a less negative potential (*). I_NS_ was larger (*), was more frequently present and peaked at less negative value (*). The I_Ca_ component was larger when measured from its beginning to its peak and when the I_Ca_ peak was compared to the symmetrical peak during repolarizing ramp. The outward current began to reincrease (“I_to_ start”) at less negative potential (*) to reach a value at the ramp peak (“I_to_”) which was smaller (*) (although it was similar when measured with respect to I_h_).

**Table 5:** Currents during 6.5 mV sec^−1^ ramp in VM and P cells

*n* 15, Param	VM cells	P cells	Δ (mV or %)
V_h_ (mV)	−80.6 ± 0.6	−81.3 ± 0.9	−0.7 mV
I_K1_ peak (pA)	965 ± 124	465 ± 57*	+107.5%
I_K1_ peak (mV)	−44.2 ± 1.7	−50.1 ± 1.2*	5.9 mV
I_NS_ (pA)	−218 ± 31 (14/15)	−45.6 ± 12.0* (10/15)	+379.3%
I_NS_ peak (mV)	−3.9 ± 3.1	−23.2 ± 5.1*	19.3 mV
I_Ca_ start (mV)	−11.6 ± 5.2	−4.4 ± 3.0	−7.2 mV
I_Ca_ (pA)	−39.9 ± 17.5 (6/15)	−20.1 ± 9.6 (4/15)	+98.5%
I_Ca_ peak (mV)	−5.1 ± 4.7	−10.5 ± 7.2	5.4 mV
Δ (pA)	−51.9 ± 6.9 (15/15)	−29.8 ± 9.3 (7/15)	+74.1%
I_to_ start (mV)	0.64 ± 2.1	−19.2 ± 3.7*	19.8 mV
I_to_ (pA)	109 ± 14	436 ± 62*	−75%
I_ramp peak_−I_h_ (pA)	812 ± 114	863 ± 71	−5.9%
I_repol_ (pA)	64.7 ± 10.8	396.4 ± 62.2*	−83.6%
I_repol_ peak (mV)	8.8 ± 2.6	−15.9 ± 4.6*	24.7 mV
I_PS_ start (mV)	8.9 ± 2.6	−5.6 ± 2.7*	14.5 mV
I_PS_ peak (pA)	197.0 ± 28.4 (15/15)	36.0 ± 19.0* (5/15)	+447.2%
I_PS_ peak (mV)	−44.4 ± 1.6	−52.1 ± 1.8	7.7 mV
I_PS_ peak−I_h_ (pA)	981 ± 124	428 ± 68*	+53.3%

*n* 15, number of cells studied; Δ (mV or %), difference in mV or percent of VM cells data with respect to P cells data; I_K1_ peak (pA), amplitude of I_K1_ peak in pA, measured as the difference from I_h_; I_K1_ peak (mV), voltage in mV at which I_K1_ peaked; I_NS_ (pA), current amplitude in pA during the negative slope region; I_NS_, peak (mV), voltage in mV of I_NS_ peak; I_Ca_ start (mV), beginning of I_Ca_ component in mV determined as the departure of current trace from I_NS_ peak; I_Ca_ (pA), amplitude of I_Ca_ component in pA as the difference between its beginning and its peak; I_Ca_ peak (mV), peak in mV of I_Ca_; Δ (pA), difference in pA between I_Ca_ peak during depolarization and minimum outward current preceding the beginning of I_PS_ on repolarization; I_to_, start (mV), voltage in mV at which the increasing outward current started at I_NS_ or I_Ca_ peaks; I_to_ (pA), amplitude of outward current in pA measured between its beginning and ramp peak; I_ramp_ _peak_−I_h_ (pA), outward current at ramp peak measured as difference from I_h_; I_repol_ (pA), amplitude in pA of the outward current between ramp peak and its smallest value prior to the beginning of I_PS_; I_repol_ peak (mV), voltage in mV at which the outward current was smallest prior to I_NS_ beginning; I_PS_ start (mV), voltage in mV at which I_PS_ began; I_PS_ peak (pA), current in pA at I_PS_ peak, measured as difference between its beginning and its peak; I_PS_ peak (mV), voltage in mV at which PS region peaked; I_PS_ peak−I_h_ (pA), current in pA measured as difference between I_NS_ peak and I_h_; V_h_ (mV), holding potential in mV; Param, parameters measured; VM cells, data from ventricular myocardial cells; P cells, data from Purkinje cells; *statistically significant difference between P and VM cells data.

During the repolarizing ramp, the decrease of outward current (I_repol_) was smaller (*) and peaked at less negative potential (*). I_PS_ (consistently present in VM but not in P cells) began at a more positive potential (*) and it was much larger (*) as it was (*) when measured with respect to I_h_ although less so. In VM cells, I_K1_ peak was similar to I_PS_ peak as it was in P cells at I_PS_ peak (or at the value at which the outward current began to decrease rapidly). This suggests that in neither tissue the Na^+^ currents contributed to I_K1_ or I_PS_ peaks.

Thus, with slow ramps, with respect to P cells, in VM cells: (1) I_K1_ peak was much larger and peaked at a less negative potential; (2) I_NS_ was larger and peaked at less negative potentials; (3) I_Ca_ component was larger; (4) the outward current enhancement prior to ramp peak (the “bulge”) was absent; (5) the outward current at ramp peak was much smaller (but less so when compared to I_h_); (6) during repolarizing ramps, the smaller I_repol_ declined much less; (7) I_PS_ was present more frequently, was larger and with a more positive beginning, and (8) I_PS_ and I_K1_ peaks were similar, both being larger than the ramp peak current.

### Slope conductance changes during slow ramps in Purkinje and myocardial cells

If, during depolarization, the gradually smaller increase in outward current is due to the inward rectification of I_K1_ channel, the slope conductance should decrease accordingly. To find out, the slope conductance was measured by superimposing small hyperpolarizing voltage pulses on the parent ramp (protocol in Fig. 6C).

In the P cell (Fig. 6D), the amplitude of the pulse current at V_h_ −80 mV decreased gradually on depolarization to reach a minimal value at −44 mV, just prior the beginning of a small NS region. During I_NS_, the pulse current reversed polarity and reincreased. With further depolarization, the pulse current decreased again, became once more negative and reincreased in amplitude. During the repolarizing ramp, similar events occurred in reverse order, including a smaller increase in slope conductance during a rather small I_PS_.

The sections of the traces labeled with a horizontal line are shown underneath at higher gain for a better visualization of pulse current changes. The arrow under the magnified trace points to a small inward component that followed the outward pulse current: such an inward component was not present during the repolarizing ramp (triangle) or in the VM cells (see below), suggesting that the brief hyperpolarizing step allowed an increased availability of sodium channels.

In the VM cell (Fig. 6E), at V_h_ the amplitude of the pulse current was larger (+72%) than in the P cell. The pulse current decreased on depolarization to become minimal at −31 mV, reversed polarity during I_NS_ and increased to a maximum at −14 mV, decreased again and then underwent a much smaller increase than in the P cell during the remainder of the ramp. During the repolarizing ramp, similar events occurred in reverse order, including an increase in slope conductance during I_PS_.

In VM cells (*n* = 11, of which 3 from the same hearts as P cells) the pulse current amplitude varied as follows: −381.7 ± 34.6 pA at V_h_ −81.8 mV, 0 pA at I_K1_ peak (−39.2 mV), +75.6 ± 19.8 pA at the −14.3 mV reversal peak during I_NS_ (which was −310.3 ± 80.3 pA, 11/11 cells), and −16.7 ± 4.9 pA at ramp peak. With repolarizing ramps, the pulse current amplitude was −107.1 ± 18.5 pA at +13.7 mV, +84.6 ± 22.0 pA at −16.1 mV during I_PS_, and 3.9 ± 3.9 pA (10/11 cells) at I_PS_ peak (which was at −42.5 ± 1.7 mV). I_K1_ and I_PS_ peaks were 1208 ± 207 and 1224 ± 210 pA, respectively.

In P cells (*n* = 21), the pulse current amplitude varied as follows. It was −190.8 ± 20.1* pA at V_h_ −88.5 mV and 0 pA at I_K1_ peak (−47.1 ± 1.5* mV). I_NS_ was 7.2 ± 4.4* pA and was present only in 3/21 cells. The pulse current amplitude was −110.0 ± 11.7* pA at the ramp peak. During the repolarizing ramp, the pulse current amplitude was −71.4 ± 6.8 pA at +17.7 mV (* with respect to −119 pA at +20.1 mV during depolarization) and 2.5 ± 2.2 pA at the potential where the final faster depolarization began (I_PS_ was present in 2/21 cells).

In the cells from the same three hearts with the same −83.3 mV V_h_, similar results were obtained in that in VM cells the pulse current amplitude at V_h_ was larger by +88.8%, the pulse current fell to 0 pA at a less negative potential, I_NS_ was +923% larger, the pulse current at ramp peak smaller by −87.5%, the reversed pulse current during I_PS_ was 73 pA (there was no I_PS_ in P cells).

Thus, with respect to P cells, in VM cells the pulse current: (1) was larger at V_h_ (+100.0%*); (2) fell to a minimum at the 7.9 mV* less negative I_K1_ peak; (3) consistently reversed and reincreased during I_NS_; (4) fell again by I_NS_ end and reincreased but much less (−84.8%* than P cells at the ramp peak; and (5) during the repolarizing ramp, the smaller VM conductance underwent the converse changes, reincreasing during I_PS_.

### Current during fast ramps in myocardial and Purkinje cells

As with the 6.5 mV sec^−1^ ramps, the Na^+^ channels would be inactivated, depolarizing and repolarizing ramps with progressively steeper slopes were applied to VM and P cells.

**Figure 7:**
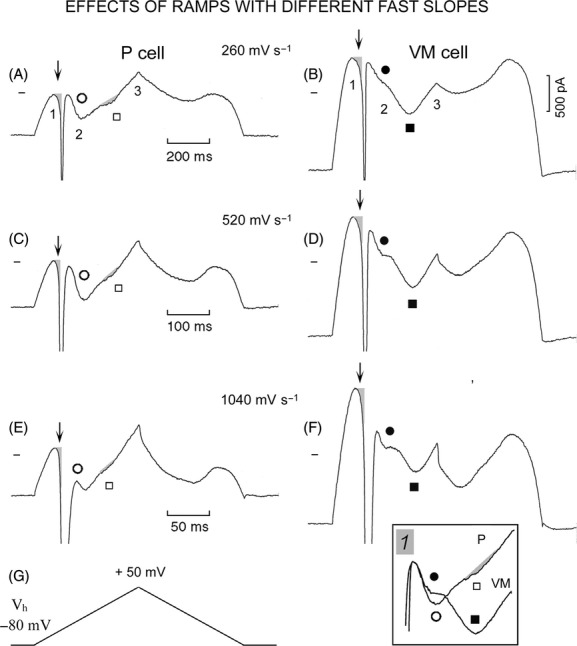
I-V relation during steep ramps in P and VM cells. The 260, 520, and 1040 mV sec^−1^ ramps were applied from V_h_ −80 to +50 mV (G) to a P cell (A, C, and E, respectively) and a VM cell (B, D, and F, respectively). The numbers 1, 2, and 3 label the peaks of I_K1_, of I_NS_, and of the ramp, respectively. I_NS_ is labeled by empty circles in the P cell and by filled circles in the VM cell. The inward component attributable to I_Ca_ is labeled by empty squares and small shaded areas in the P cell, and by filled squares in the VM cell. In VM cells, I_Ca_ was measured from its beginning (taken as the point at which the slope of I_NS_ met the backward extrapolation of I_Ca_) and I_Ca_ peak. The downward vertical arrows point to the slowly increasing inward current (shaded areas) preceding the activation of I_Na1_. In inset 1, the traces from C and D were superimposed by the end of I_Na1_ inactivation. In both cells, I_Na1_ was cut off by the saturation of the amplifier at −10 nA.

In Figure 7, 260 mV sec^−1^ ramp, with respect to the P cell (Fig. 7A), in the VM cell (Fig. 7B) at point 1 I_K1_ peak was much larger and less negative. In both P and VM cells, immediately after I_K1_ peak, a slowly increasing I_Na3_ (shaded areas labeled by downward arrows) preceded the activation of I_Na1_, as expected from the more negative threshold of I_Na3_ (see Figs. 1 and 3). In the P cell, I_NS_ (empty circle) peaked at point 2 and was followed by an increasing outward current. A very shallow inward component, peaking at +12 mV, appeared as an “indentation” on the increasing outward current (empty square and small shaded area). In the VM cell, I_NS_ (filled circle) was smaller and was followed by a large inward component (filled square) which peaked at +17 mV, as expected from a larger I_Ca_ component.

Similar but not identical results were obtained during the 520 mV sec^−1^ (Fig. 7C and D) and 1040 mV sec^−1^ (Fig. 7E and F) ramps. I_K1_ peak increased in magnitude with the steeper ramps both in P and VM cell, still being much smaller in the P cell. In both cells, the steeper ramp slope caused I_Na1_ to inactivate closer to the end of I_NS_. As usual, the outward current at ramp peak (point 3) was larger than I_K1_ peak (point 1) in the P cell (Fig. 7C and E) whereas it was smaller in the VM cell (Fig. 7D and F). I_NS_ of the P cell (Fig. 7C) and that of the VM cell (Fig. 7D) were superimposed by the end of I_Na1_ inactivation in Figure 7 inset 1. While I_NS_ was larger in the P cell, the subsequent I_Ca_ component was much larger (filled square) in the VM cell.

On repolarization, in the P cell the outward current decreased more and peaked at more negative values than in the VM cell. In both cells, on repolarization with steeper ramps the decrease in outward current was faster initially and it was larger. Also, I_PS_ started from a less outward value and, on that account, I_PS_ peak became smaller than the I_K1_ peak.

As shown in Table 6, during the 260 mV sec^−1^ ramp with respect to P cells, in VM cells I_K1_ peak was larger (*) and less negative (*), I_NS_ was smaller and its peak was less negative (*). I_Na3_ amplitude was somewhat greater, I_Na1_ threshold was less negative (*), I_Na1_ amplitude was similar (there was I_Na1_ in 14/17 VM cells and in 8/17 P cells).

**Table 6:** Currents during the 260 mV sec^−1^ ramp in VM and P cells

*n* 17, Param	VM cells	P cells	Δ (mV or %)
V_h_ (mV)	−80.6 ± 0.6	−81.8 ± 1.0	−1.2 mV
I_K1_ peak (pA)	913 ± 101	477 ± 56.8*	+91.4%
I_K1_ peak (mV)	−52.1 ± 1.0	−57.3 ± 1.0*	5.2 mV
I_NS_ (pA)	−216.6 ± 26.3	−340.5 ± 44.3*	−36.3 %
I_NS_ peak (mV)	−14.0 ± 1.6	−26.5 ± 1.5*	12.5 mV
I_Na3_ (pA)	−941 ± 119 (14/17)	−733 ± 89 (8/17)	+28.3%
I_Na1_Th (mV)	−40 ± 1.5	−47.3 ± 1.8*	−7.2 mV
I_Na1_ (pA)	−7298 ± 842 (14/17)	−6978 ± 850 (8/17)	+4.5%
End I_Na1_ (pA)	910 ± 109 (14/17)	324 ± 64* (8/17)	+180.8%
End I_Na1_ (mV)	−34.2 ± 1.5	−39.5 ± 1.5*	5.3 mV
I_Ca_ start (mV)	−9.2 ± 2.0	−15 ± 1.0*	5.8 mV
I_Ca_ (pA)	−108.3 ± 24.2 (15/17)	−19.6 ± 11.6* (3/17)	+452.5%
I_Ca_ peak (mV)	14.0 ± 0.9	−3.3 ± 3.7*	17.3 mV
I_to_ start (mV)	13.7 ± 0.9	−21.3 ± 2.8*	35 mV
I_to_ (pA)	206.0 ± 25.3 (17/17)	1270 ± 166* (17/17)	−83.7%
I_ramp peak_−I_h_ (pA)	783 ± 78 (17/17)	1373 ± 166* (17/17)	−42.9%
I_repol_ (pA)	123.5 ± 22	967 ± 139*	−87.2%
I_repol_ peak (mV)	7.2 ± 3.2	−15.9 ± 1.6*	23.1 mV
I_PS_ start (mV)	3.3 ± 3.2	−17.2 ± 1.7*	20.4 mV
I_PS_ (pA)	214 ± 29.8 (17/17)	64.6 ± 13.2* (15/17)	+231.2%
I_PS_ peak (mV)	−45.2 ± 1.6	−46.7 ± 2.9	1.5 mV
I_PS_ peak−I_h_ (pA)	895 ± 103 (17/17)	395 ± 58* (15/17)	+126.5%

End I_Na1_ (pA), Amplitude in pA of the current at the end of I_Na1_ inactivation; End I_Na1_ (mV), voltage in mV of the current at the end of I_Na1_ inactivation; V_h_ (mV), holding potential in mV; Param, parameters measured; VM cells, data from ventricular myocardial cells; P cells, data from Purkinje cells; I_Na1_ Th (mV), threshold potential in mV of I_Na1_; I_Na3_ (pA), amplitude in pA of I_Na3_ measured as the difference between its peak and the end of the step; I_Na1_ (pA), amplitude in pA of I_Na1_; Numbers in parenthesis (e.g., 14/17), number of cells in which the parameter was present over the total number of cells studied; *statistically significant difference between P and VM cells data. Other explanations as in the legend of Table 5.

The I_Ca_ component was larger (*) (there was a measurable I_Ca_ component in15/17 VM cells and in 3/17 P cells). The outward current between I_Ca_ and ramp peaks (“I_to_”) began at a more positive potential (*) and was smaller (*). When the ramp peak current was measured as the difference from I_h_, I_to_ was smaller (*) but less so, due to the larger I_K1_ upon which the ramp peak current was superimposed.

The I_K1_ peak was similar to the current at the end of I_Na1_ inactivation both in the VM cells (0.003%) and in the eight P cells in which I_Na1_ was present (I_K1_ peak 389.1 pA and at the end of I_Na1_ 324.6 pA, difference not statistically significant). These results are consistent with the elimination of the slow inactivation of I_Na3_ by I_Na1_ in both tissues. At the end of I_Na1_, the voltage was less negative in VM cells (*), reflecting the less negative I_Na1_ threshold (*).

In VM cells, with respect to the 260 mV sec^−1^ ramp, the 520 (Table 7) and 1040 mV sec^−1^ (Table 8) *depolarizing* ramps induced the following changes, respectively: peak I_K1_ +5.4% and +18.5%, I_Na3_ +75.0%* and +132%*, I_Na1_ +39.3% and +53.0%*, I_NS_ +28.3% and +86.9%,* I_Ca_ component +10.0% and +3.0%, “I_to_” +8.2% and +7.8%, and I_ramp peak_−I_h_, +5.1% and +12.8%.

**Table 7:** Currents during the 520 mV sec^−1^ ramp in VM and P cells

*n* 17, Param	VM cells	P cells	Δ (mV or %)
V_h_ (mV)	−80.6 ± 0.5	−81.8 ± 0.9	−0.7 mV
I_K1_ peak (pA)	963 ± 108	518 ± 57*	+46.2%
I_K1_ peak (mV)	−52.3 ± 0.7	−58.3 ± 0.7*	6 mV
I_NS_ (pA)	−278 ± 36	−546 ± 48*	−49.0%
I_NS_, peak	−18.1 ± 2.6	−22.4 ± 1.6	4.3 mV
I_Na3_ (pA)	−1458 ± 170 (17/17)	−1352 ± 175 (14/17)	+7.8%
I_Na1_Th (mV)	−39.2 ± 1.2	−46.8 ± 1.2*	7.6 mV
I_Na1_ (pA)	−8373 ± 553 (17/17)	−9439 ± 352 (14/17)	−11.9%
End I_Na1_ (pA)	837 ± 113	381 ± 69*	+119.6%
End I_Na1_ (mV)	−32.6 ± 1.2	−38.5 ± 1.0*	5.9 mV
I_Ca_, start (mV)	−4.9 ± 1.9	−11.5 ± 0.5*	6.6 mV
I_Ca_ (pA)	−119.2 ± 31.7 (16/17)	−16.4 ± 14.1* (2/17)	+626.8%
I_Ca_ peak (mV)	17.4 ± 0.9	−3.5 ± 7.5	20.9 mV
I_to_, start (mV)	10.3 ± 3.4	−17.1 ± 2.0* (17/17)	27.4 mV
I_to_ (pA)	223 ± 27.0	1478 ± 173* (17/17)	−84.9%
I_ramp peak_−I_h_ (pA)	823 ± 88	1405 ± 167	−41.4%
I_repol_ (pA)	198 ± 32	1169 ± 144*	−83.0%
I_repol_ peak (mV)	3.1 ± 3.6	−16.6 ± 2.0*	19.4 mV
I_PS_, start (mV)	0.2 ± 3.7	−17.1 ± 1.8*	17.3 mV
I_PS_ (pA)	219 ± 34	122 ± 20	79.5%
I_PS_ peak (mV)	−45.3 ± 1.7	−49.8 ± 1.0*	4.5 mV
I_PS_ peak−I_h_ (pA)	850 ± 105	349 ± 55*	+143.5%

*n*, number of cells studied; Δ (mV or%), difference in mV or percent of VM cells data with respect to P cells data; I_K1_ peak (pA), amplitude of I_K1_ peak in pA, measured as the difference from I_h_; I_K1_ peak (mV), voltage in mV at which I_K1_ peaked; I_NS_ (pA), current amplitude in pA during the negative slope region; I_NS_, peak (mV), voltage in mV of I_NS_ peak; I_Na1_ Th (mV), voltage at which I_Na1_ began; I_Na1_ (pA), amplitude in pA of I_Na1_; I_Ca_ start (mV), beginning of I_Ca_ component in mV determined as the departure of current trace from I_NS_ peak; I_Ca_ (pA), amplitude of I_Ca_ component in pA as the difference between its beginning and its peak; I_Ca_ peak (mV), peak in mV of I_Ca_; I_to_, start (mV), voltage in mV at which the increasing outward current started at I_NS_ or I_Ca_ peaks; I_to_ (pA), amplitude of outward current in pA measured between its beginning and ramp peak; I_ramp_ _peak_−I_h_ (pA), outward current at ramp peak measured as difference from I_h_; I_repol_ (pA), amplitude in pA of the outward current between ramp peak and its smallest value prior to the beginning of I_PS_; I_repol_ peak (mV), voltage in mV at which the outward current was smallest prior to I_NS_ beginning; I_PS_ start (mV), voltage in mV at which I_PS_ began; I_PS_ (pA), current in pA at I_PS_ peak, measured as difference between its beginning and its peak; I_PS_ peak (mV), voltage in mV at which PS region peaked; I_PS_ peak−I_h_ (pA), current in pA measured as difference between I_NS_ peak and I_h_; End I_Na1_ (pA), amplitude in pA of the current at the end of I_Na1_ inactivation; End I_Na1_ (mV), voltage in mV of the current at the end of I_Na1_ inactivation; V_h_ (mV), holding potential in mV; Param, parameters measured; VM cells, data from ventricular myocardial cells; P cells, data from Purkinje cells; I_Na3_ (pA), amplitude in pA of I_Na3_ measured as the difference between its peak and the end of the step; Numbers in parenthesis (e.g., 17/17), number of cells with the parameter present over the total number of cells studied; *statistically significant difference between P and VM cells data.

**Table 8:** Currents during the 1040 mV sec^−1^ ramp in VM and P cells

*n* 17, Param	VM cells	P cells	Δ (mV or %)
V_h_ (mV)	−80.6 ± 0.5	−81.8 ± 0.9	1.2 mV
I_K1_ peak (pA)	1082 ± 113	614 ± 60*	+76.2%
I_K1_ peak (mV)	−52.2 ± 0.7	−58.0 ± 0.7*	5.8 mV
I_NS_ (pA)	−405 ± 57	−710 ± 74*	−42.9%
I_NS_ peak (mV)	−16.2 ± 2.6	−19.8 ± 1.4	3.6 mV
I_Na3_ (pA)	−1986 ± 132	−1645 ± 199	+20.7%
I_Na1_ Th (mV)	−38.3 ± 1.1	−47.1 ± 0.9*	8.8 mV
I_Na1_ (pA)	−9197 ± 393 (17/17)	−9491 ± 280 (16/17)	−3.09%
End I_Na1_ (pA)	863 ± 105 (17/17)	351 ± 69* (16/17)	+145.8%
End I_Na1_ (mV)	−27.9 ± 1.5	−30.8 ± 4.4	2.9 mV
I_Ca_ start (mV)	−0.6 ± 1.7	−8.0 ± 0.0	7.2 mV
I_Ca_ (pA)	−111.6 ± 28.6 (14/17)	−19.5 ± 18.0* (2/17)	+472%
I_Ca_ peak (mV)	20.9 ± 1.1	−4.0 ± 1*	24.9 mV
I_to_ start (mV)	14.0 ± 4.0	−14.1 ± 3.25*	28.1 mV
I_to_ (pA)	222 ± 28	1776 ± 187*	−87.5%
I_ramp peak_−I_h_ (pA)	884 ± 85	1658 ± 172	−46.6%
I_repol_ (pA)	396 ± 50	1522 ± 158*	−73.9%
I_repol peak_ (mV)	10 ± 3.3	−16.7 ± 2.0*	26.7 mV
I_PS_ start (mV)	5.4 ± 3.6	−18.9 ± 1.9*	24.3 mV
I_PS_ (pA)	223 ± 40	163 ± 24 (15/17)	+36.8%
I_PS_ peak (mV)	−47.2 ± 1.7	−50.4 ± 1.0	3.2 mV
I_PS_ peak−I_h_ (pA)	733 ± 100	244 ± 47*	+200.4%

*n*, number of cells studied; Δ (mV or%), difference in mV or percent of VM cells data with respect to P cells data; I_K1_ peak (pA), amplitude of I_K1_ peak in pA, measured as the difference from I_h_; I_K1_ peak (mV), voltage in mV at which I_K1_ peaked; I_NS_ (pA), current amplitude in pA during the negative slope region; I_NS_, peak (mV), voltage in mV of I_NS_ peak; I_Na1_ Th (mV), voltage at which I_Na1_ began; I_Na1_ (pA), amplitude in pA of I_Na1_; I_Ca_ start (mV), beginning of I_Ca_ component in mV determined as the departure of current trace from I_NS_ peak; I_Ca_ (pA), amplitude of I_Ca_ component in pA as the difference between its beginning and its peak; I_Ca_ peak (mV), peak in mV of I_Ca_; I_to_, start (mV), voltage in mV at which the increasing outward current started at I_NS_ or I_Ca_ peaks; I_to_ (pA), amplitude of outward current in pA measured between its beginning and ramp peak; I_ramp_ _peak_−I_h_ (pA), outward current at ramp peak measured as difference from I_h_; I_repol_ (pA), amplitude in pA of the outward current between ramp peak and its smallest value prior to the beginning of I_PS_; I_repol_ _peak_ (mV), voltage in mV at which the outward current was smallest prior to I_NS_ beginning; I_PS_ start (mV), voltage in mV at which I_PS_ began; I_PS_ (pA), current in pA at I_PS_ peak, measured as difference between its beginning and its peak; I_PS_ peak (mV), voltage in mV at which PS region peaked; I_PS_ peak−I_h_ (pA), current in pA measured as difference between I_PS_ peak and I_h_; End I_Na1_ (pA), Amplitude in pA of the current at the end of I_Na1_ inactivation; End I_Na1_ (mV), voltage in mV of the current at the end of I_Na1_ inactivation; V_h_ (mV), holding potential in mV; Param, parameters measured; VM cells, data from ventricular myocardial cells; P cells, data from Purkinje cells; I_Na3_ (pA), amplitude in pA of I_Na3_ measured as the difference between its peak and the end of the step; Numbers in parenthesis (e.g., 17/17), number of cells in which the parameter was present over the total number of cells studied; *statistically significant difference between P and VM cells data. Other explanations as in the legend of Table 7.

In P cells, with respect to the 260 mV sec^−1^ ramp, the 520 (Table 7) and 1040 mV sec^−1^ (Table 8) ramps induced the following changes, respectively: peak I_K1_ +8.5% and +28.7%, I_Na3_ +291.8%* and +376.8%*, I_Na1_ +187.5%* and +189.0%*, I_NS_ +60.5%* and +108.8%*, “I_to_” +16.3% and +39.8%*, and I_ramp peak_−I_h_, +2.3% and +20.7%*.

Thus, in both VM and P cells the faster ramps increased I_K1_ peak, I_Na3_, I_Na1_, I_NS,_ and I_ramp peak_ when measured either from its beginning during the ramp or from I_h_. The increase in I_Na3_ and in I_NS_ was larger in P cells, consistent with I_Na3_ role in I_NS_.

As for the *repolarizing* ramps, with respect to P cells, in VM cells during the 260 mV sec^−1^ ramp (Table 6), the decreasing outward current was smaller (*) and peaked at a more positive potential (*). I_PS_ began at a less negative potential (*) and was larger (*). It was larger also when measured as the difference from I_h_ (*).

In VM cells, with respect to the 260 mV sec^−1^ repolarizing ramp, the 520 (Table 7) and 1040 mV sec^−1^ (Table 8) repolarizing ramps induced the following changes, respectively: I_repol_ larger by +60.3% and by +220%*, the difference in initiation of I_PS_ 3.1 and 2.1 mV, difference in voltage of I_PS_ peak 0.1 and 2 mV, amplitude of I_PS_ peak +0.2% and +4.2% and, when compared to I_h_, −5.0% and −18.1%. Therefore, on repolarization the decrease of I_repol_ (but not I_PS_) was sensitive to the repolarizing ramp slope.

In P cells, with respect to the 260 mV sec^−1^ repolarizing ramp, the 520 (Table 7) and 1040 mV sec^−1^ (Table 8) repolarizing ramps induced the following changes, respectively: I_repol_ larger by 20.8% and by 57.3%*, difference in I_PS_ initiation 0.1 and 1.8 mV, difference in voltage of I_PS_ peak −3.1 and −3.7 mV, amplitude of I_PS_ peak +88.8%* mV and +152.3%* and, when measured from I_h_, −11.6% and −38.2%*.

Therefore, in P cells I_Na3_ and I_NS_ became greater with faster depolarizing ramps, suggesting that sodium currents play a larger role in NS region of P cells than in that of VM cells. Also, during repolarizing ramp, the outward current decreased more with faster ramps in both P and VM cells. With faster ramps, starting from a lower value, I_PS_ increased in P cells, but decreased when measured as the difference from I_h_.

### Differences in I_NS_ in Purkinje versus myocardial cells

A decrease of V_h_ from −90 mV to −60 mV markedly decreased the amplitude of I_NS_ in P cells (Rota and Vassalle 2003; Vassalle et al. 2007; present results), but not in VM cells as measured using the sustained current at the end of depolarizing steps (Fig. 4). The finding suggests that the Na^+^ current may play a predominant role in the mechanisms underlying I_NS_ in P but not in VM cells. This was tested by applying ramps from different V_h_.

**Figure 8:**
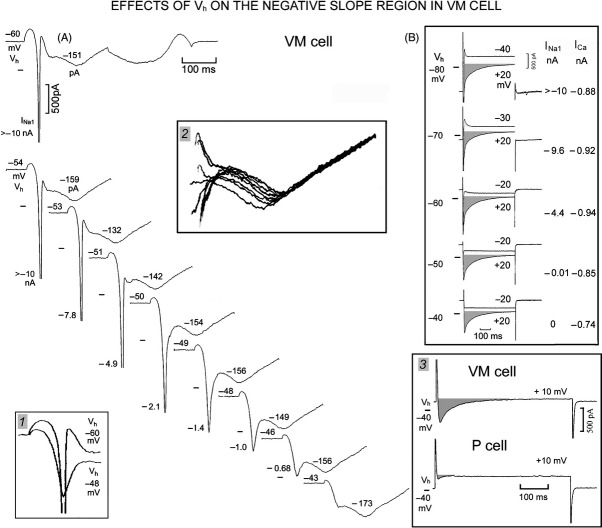
Persistence of NS region and of I_Ca_ with ramps from lower holding potentials in VM cells. In A, 520 mV sec^−1^ ramps were applied from the V_h_ indicated above I_h_ (−60 to −43 mV in 1 mV decrements; not all traces shown). I_Na1_ amplitude in nA is indicated next to the tip of I_Na1_ traces. In inset 1, the traces with V_h_ −60 and −48 mV have been superimposed. The amplitude in pA of I_Ca_ component (measured from its beginning to its peak) is indicated by the number above the traces. In inset 2, the superimposed traces show that I_Ca_ component was not affected by gradually smaller V_h_. In B (from a different heart), depolarizing steps were applied from gradually lower V_h_ and the currents were superimposed at the indicated voltages. The amplitudes of I_Na1_ and of I_Ca_ (shaded area) are indicated in nA at the right hand of the traces. In inset 3, the voltage step was applied from V_h_ −40 to +10 mV in a VM and P cell.

In Figure 8A, in a VM cells, V_h_ was progressively decreased by 1 mV and 520 mV sec^−1^ ramps were applied from V_h_ indicated above the I_h_ trace (−60 to −43 mV). At V_h_ −60 mV (top *A* trace), I_Na1_ was truncated at −10 nA and I_NS_ was followed by I_Ca_ component (−151 pA, as indicated above the trace) peaking at +22 mV. With V_h_ −54 mV, I_Na1_ was still larger than −10 nA, but beginning with V_h_ –53 mV, I_Na1_ gradually decreased as indicated in nA next to the tip of I_Na1_ traces. Also, as I_Na1_ became much smaller, the activation and inactivation of the inward transient became slower, as illustrated in Figure 8 inset 1 by the superimposed V_h_ −60 and −48 mV traces. At V_h_ −43 mV (bottom trace), there was no apparent I_Na1_ and I_NS_ had an amplitude of −462 pA, a duration of 36 msec and a voltage range between −40 and −12 mV. Over the range of V_h_ tested, the I_Ca_ component peaked at ∼+20 mV and its amplitude remained at ∼−150 pA (see numbers above the I_Ca_ component traces and the superimposed traces in Figure 8 inset 2).

That the inward component positive to I_NS_ was due to I_Ca_ is supported by the results obtained with depolarizing steps applied from V_h_ −80, −70, −60, −50, and −40 mV (Fig. 8B). The amplitudes of I_Na1_ and of that of I_Ca_ (shaded areas) are indicated, respectively, in nA at the right hand of traces. I_Na1_ decreased gradually with the less negative V_h_ and disappeared with V_h_ −50 and −40 mV. Instead, the slowly inactivating current persisted even with V_h_ of −40 mV (albeit somewhat decreased) as expected for I_Ca_ (Isenberg and Klöckner 1982). In Figure 8 inset 3, depolarizing steps were applied from V_h_ −40 to +10 mV and show that I_Ca_ was far larger in the VM than in the P cell, as usual.

The results are consistent with Na^+^ currents playing little role in I_NS_ of VM cells and also with I_Ca_ underlying the inward component that peaked at ∼+20 mV (as well as the small indentation over a similar voltage range in P cells).

In Table 9, in VM cells fast ramps were applied from V_h_ −80 and −50 mV with the following changes. I_K1_ peak decreased (*) as the K1 channel rectified inwardly at lower V_h_ before the ramp was applied. I_NS_ was not affected whereas I_Na1_ markedly decreased (*). The I_Ca_ component was similar. The ramp peak current (I_to_) increased (the ramp peak voltage being more positive), although it was smaller when measured as I_ramp peak_−I_h_ (*) due to the inward shift of the current with less negative V_h_. I_PS_ and its peak voltage were similar. However, I_PS_ peak measured relative to I_h_ was 820 pA in control and −5.0 pA* with the less negative V_h_, reflecting the inward shift of I_K1_ with V_h_ −50 mV. In 6 of 7 of these experiments, V_h_ was also decreased by 1–2 mV with the results similar to those illustrated in Figure 8A.

**Table 9:** Less negative V_h_ of fast ramps markedly decreases I_NS_ and I_PS_ in P but not in VM cells

Param	VM cells *n* 7	VM cells	P cells *n* 4	P cells
V_h_ (mV)	−80 ± 0	−50 ± 0	−82.5 ± 2.5	−55.0 ± 2.8
I_K1_ peak (pA)	999 ± 223	92 ± 19*	270 ± 57	53.0 ± 15.5*
I_K1_ peak (mV)	−47.7 ± 4.4	−41.5 ± 2.8	−60 ± 3.4	−47.0 ± 2.8*
I_NS_ (pA)	−388 ± 75	−246 ± 46	−261 ± 91	−10.5 ± 6.5 (2/4) *
I_NS_ peak (mV)	−9.3 ± 4.7	−22.7 ± 3.4	−26.3 ± 3.0	−35.8 ± 6.3 (2/4)
I_Na1_ (pA)	−7988 ± 1170	−298 ± 87*	−9334 ± 665	−123 ± 123 (1/4) *
I_Ca_ start (mV)	−7.5 ± 4.7	−7.1 ± 5.8	0 ± 0	0 ± 0
I_Ca_ (pA)	−184 ± 52	−183 ± 77	0 ± 0	0 ± 0
I_Ca_ peak (mV)	15.5 ± 10.3	13.6 ± 5.4	0 ± 0	0 ± 0
I_to_ start (mV)	5.6 ± 7.8	14.2 ± 4.6	−15.4 ± 12	−6.8 ± 17.7
I_to_ peak (pA)	341 ± 87	542 ± 132	1586 ± 579	1789 ± 667
I_ramp peak_−I_h_ (pA)	842 ± 154	284 ± 98*	1597 ± 565	1826 ± 677
I_repol_ (pA)	192 ± 53	511 ± 104*	1420 ± 582	1831 ± 679
I_repol peak_ (mV)	8.6 ± 7.5	−5.6 ± 5.2	−31.1 ± 5.3	−30.9 ± 9.2
I_PS_ start (mV)	5.7 ± 8.0	4.4 ± 4.5	−28.8 ± 6.8	0 ± 0*
I_PS_ peak (pA)	214 ± 61	218 ± 63	36.0 ± 20 (3/4)	0 ± 0
I_PS_ peak (mV)	−39.2 ± 6.0	−41 ± 5.0	−50.5 ± 5.0	0 ± 0*
I_PS_−I_h_ (pA)	820 ± 198	−5.0 ± 15.2*	213 ± 81 (3/4)	24.6 ± 15*

*n*, number of cells studied; I_K1_ peak (pA), amplitude of I_K1_ peak in pA, measured as the difference from I_h_; I_K1_ peak (mV), voltage in mV at which I_K1_ peaked; I_NS_ (pA), current amplitude in pA during the negative slope region; I_NS_, peak (mV), voltage in mV of I_NS_ peak; I_Na1_ (pA), amplitude in pA of I_Na1_; I_Ca_ start (mV), beginning of I_Ca_ component in mV determined as the departure of current trace from I_NS_ peak; I_Ca_ (pA), amplitude of I_Ca_ component in pA as the difference between its beginning and its peak; I_Ca_ peak (mV), voltage in mV at which I_Ca_ peaked; I_to_, start (mV), voltage in mV at which the increasing outward current started at I_NS_ or I_Ca_ peaks; I_to_ peak (pA), amplitude of outward current in pA measured between its beginning and ramp peak; I_ramp peak_−I_h_ (pA), outward current at ramp peak measured as difference from I_h_; I_repol_ (pA), amplitude in pA of the outward current between ramp peak and its smallest value prior to the beginning of I_PS_; I_repol_ peak (mV), voltage in mV at which the outward current was smallest prior to I_NS_ beginning; I_PS_ start (mV), voltage in mV at which I_PS_ began; I_PS_ peak (pA), current in pA at I_PS_ peak, measured as difference between its beginning and its peak; I_PS_ peak (mV), voltage in mV at which PS region peaked; I_PS_ peak−I_h_ (pA), current in pA measured as difference between I_PS_ peak and I_h_; V_h_ (mV), holding potential in mV; Param, parameters measured; VM cells, data from ventricular myocardial cells; P cells, data from Purkinje cells; *statistically significant difference between the data at the two V_h_ values either in VM or P cells.

In Table 9, in P cells, the same procedure decreased I_K1_ peak (*), I_NS_ (*), and I_Na1_ (*). I_Ca_ was not measurable (only small indentations) either in control or at less negative V_h_. “I_to_” and I_ramp peak_–I_h_ increased but not significantly. Furthermore, I_PS_ was smaller than in VM cells by 83.1% with V_h_ −80 mV and was not present with V_h_ −50 mV.

In Table 10, with V_h_ −80 mV during the step to ∼+20 mV I_Ca_ was larger in VM cells (328%*) and did not decrease with gradually lower V_h_ in either VM cells (−24.8%, −6.5%, +17.5% and −29.6%, respectively) nor in P cells. However, in the latter tissue the I_Ca_ values varied irregularly, possibly due to the far fewer of P cells displaying it. Instead, as reported in Table 2, during depolarizing steps from V_h_ −70, −60, −50, and −40 mV, in VM cells I_Na1_ decreased by −3.4%, −30.3%, −75.6%, and −99.3%, and in P cells by −3.7%, −20.1%, −65.9%, −93.5%, respectively.

**Table 10:** I_Ca_ during depolarizing steps from different V_h_

V_h_ (mV)	Param	VM cells	P cells
−80	I_Ca_ peak (mV)	+19.4 ± 0.5	+18.8 ± 1.2
	I_Ca_ (pA)	−415 ± 101 (18/18)	−96.8 ± 44.6* (6/18)
	τ_f_ (msec)	17.9 ± 7.9	11.4 ± 6.3
	τ_s_ (msec)	107.8 ± 15.1	157 ± 61.2*
−70	I_Ca_ peak (mV)	19.4 ± 0.6	19.4 ± 0.6
	I_Ca_ (pA)	−312 ± 71 (15/16)	−18.9 ± 10.7* (3/16)
−60	I_Ca_ peak (mV)	19.3 ± 0.7	20.0 ± 0.0
	I_Ca_ (pA)	−388 ± 73 (15/15)	−8.8 ± 6.1* (2/15)
−50	I_Ca_ peak (mV)	20.0 ± 0.9	16.9 ± 1.5
	I_Ca_ (pA)	−488 ± 76 (16/16)	−9.1 ± 34.7* (5/16)
−40	I_Ca_ peak (mV)	20.0 ± 0.0	18.3 ± 1.6
	I_Ca_ (pA)	−292 ± 67 (10/10)	−87.3 ± 41.3* (3/6)

I_Ca_ peak (mV), voltage in mV at which the I_Ca_ was largest; I_Ca_ (pA), amplitude of I_Ca_ in pA measured from its peak to the end of the step; V_h_ (mV), holding potential in mV; Param, parameters measured; VM cells, data from ventricular myocardial cells; P cells, data from Purkinje cells; τ_f_ (msec) and τ_s_ (msec), fast and slow time constants, respectively, of I_Ca_ inactivation; Numbers in parenthesis (e.g., 18/18), number of cells in which I_Ca_ was present over the total number of cells studied; *statistically significant difference between P and VM cells data.

Thus, with less negative V_h_, I_Na1_ markedly decreased in both tissues during both ramps and depolarizing steps. In VM cells, I_NS_ and I_PS_ regions persisted even at V_h_ −50 mV. Instead, in P cells with V_h_ −80 mV, I_PS_ was much smaller than I_NS_ and, with V_h_ −50 mV, I_NS_ was markedly diminished and I_PS_ abolished. Furthermore, in VM cells I_Ca_ was consistently present with V_h_ −80 mV and did not decrease with V_h_ −50 mV. In P cells, I_Ca_ was either not apparent or it was a small inward indentation on the outward current.

### The transient outward current I_to_ in Purkinje and myocardial cells

Some of differences between currents in P and VM cells in the −20 to +40 mV range (see Fig. 2) were analyzed by applying the depolarizing steps from V_h_ −80 and V_h_ −40 mV in the absence and presence of I_to_ blocker 4-aminopyridine.

**Figure 9:**
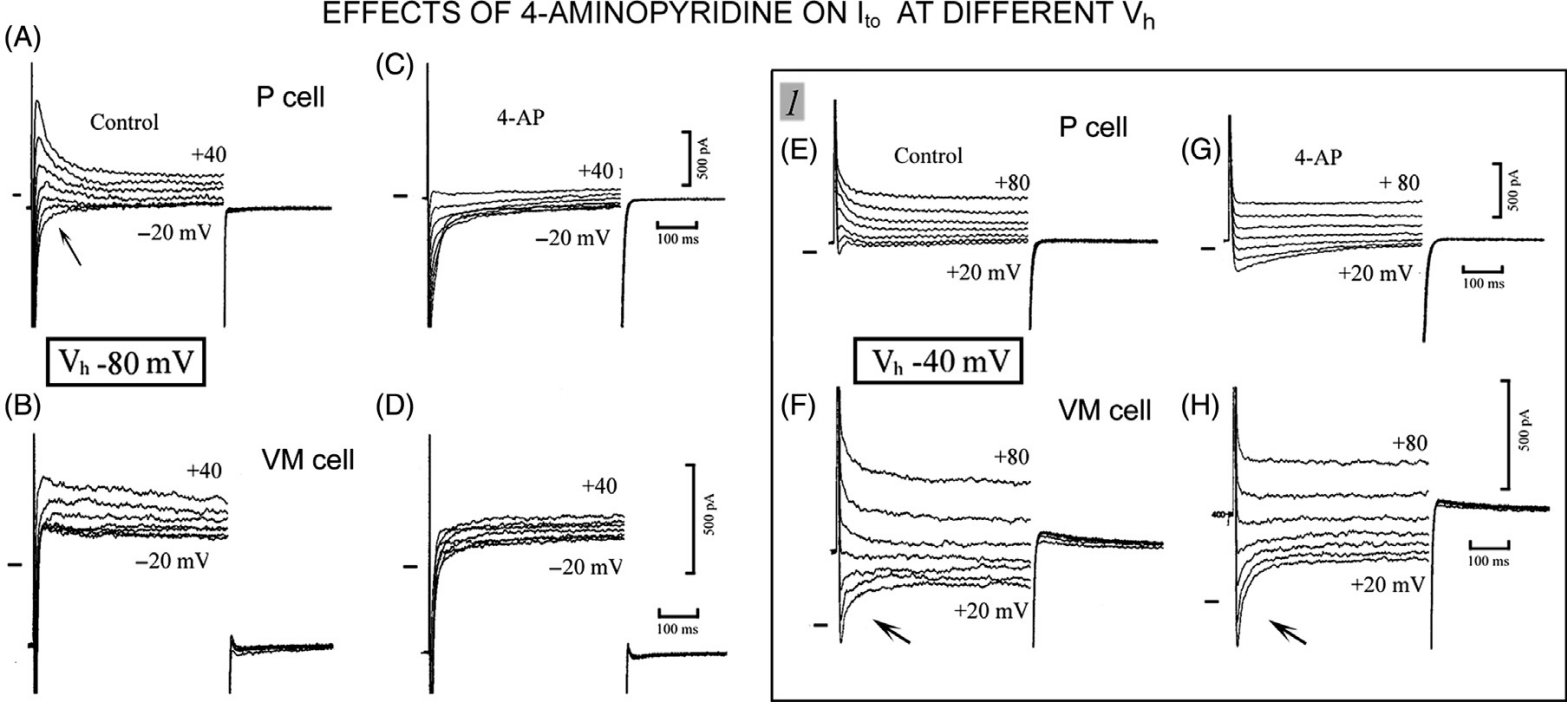
I_to_ in P and VM cells. In A–D, steps were applied from V_h_ −80 to the −20/+40 mV range. In P cell, the arrow points to slowly decaying I_Na2_ during the −20 mV step (A). The currents in a VM cell taken from a different heart are shown in B. The procedure was repeated in the presence of 4-AP in P (C) and VM cell (D). In inset 1, steps were applied from V_h_ −40 to +80 mV in P (E) and VM cell (F), where the arrow points to the current recorded at +20 mV. The procedure was repeated in the presence of 4-AP in P (G) and VM cell (H), where the arrow points the current recorded at +20 mV. VM cells from the same heart as in Fig. 2.

In Figure 9A, in P cell, during the step from V_h_ −80 to −20 mV, I_Na2_ slowly decayed (arrow). A small outward peak appeared at the beginning of the step to 0 mV and progressively increased in size with more positive steps. In Figure 9B, in VM cell from a different heart, no decaying inward I_Na2_ was apparent and, instead, a smaller outward current grew in amplitude over the range −20 to +40 mV. The sustained current at the end of the steps was smaller than in P cell (note the different current calibration).

In the presence of 4-AP in the P cell (Fig. 9C) the peak I_to_ was abolished, the decaying I_Na2_ was larger at negative values, and the sustained current was reduced. In the VM cell (Fig. 9D), 4-AP abolished the peak I_to_ and reduced the sustained current. An inward transient was present at −20 mV which increased at +20 mV. Thus, at −20 mV in P cells the inward current was much larger, inactivated more slowly than the current in VM cell, and decreased markedly at + 20 mV.

In Figure 9 inset 1, V_h_ was decreased to −40 mV in order to inactivate I_Na2_ (Vassalle et al. 2007; Bocchi and Vassalle 2008; present results) but not I_Ca_ (Isenberg and Klöckner 1982). At V_h_ −40 mV, I_to_ channel is partially inactivated, mid-inactivation voltage being ∼−35 mV (Dumaine and Cordeiro 2007). In the P cell (Fig. 9E), the inward transients were markedly reduced and peak I_to_ was decreased. In the VM cell (Fig. 9F), inward transients were present during the +20 (arrow), +30, and +40 mV steps and I_to_ was present during the +60, +70, and +80 mV steps.

In the presence of 4-AP, in the P cell (Fig. 9G), I_to_ was eliminated and the small slowly decaying inward component was largest at 0 mV. In the VM cell (Fig. 9H), in the presence of 4-AP, I_to_ peak was eliminated and the sustained current was smaller. I_Ca_ (arrow) was largest at +30 mV and reversed between +60 and +70 mV.

Thus, I_to_ patterns were distinctly different in that in the P cell I_to_ peak was larger and declined more by the end of the step. Also, I_to_ block by 4-AP unmasked inward currents with voltage range, magnitude, and speed of inactivation consistent with I_Na2_ in P cell and with I_Ca_ in the VM cell. With V_h_ −40 mV, in the P cell I_Na2_ was small and in the presence of 4-AP the current decreased slowly. In the VM cell, with V_h_ −40 mV, I_Ca_ was larger in the presence of 4-AP and inactivated quickly.

In Table 11, with V_h_ −80 mV and test steps to +40 mV, in P cells I_to_ when measured as the difference between its peak and sustained current at end of 500 msec steps was larger (*) as it was when measured as the difference between I_to_ peak and I_h_ (*). I_to_ decreased from its peak to the end of the step by −27.1% in VM and by −45.1% in P cells, the slow time constant of inactivation being smaller in VM cells (*).

**Table 11:** I_to_ in VM and P cells during depolarizing steps from different V_h_

V_h_ to test step (mV)	Param	VM cells	P cells
−80 to +40	I_to_ (pA)	394 ± 136 (12/18)	914 ± 168* (18/18)
	τ_f_ (msec)	8.8 ± 1.6	13.4 ± 2.2
	τ_s_ (msec)	59.4 ± 9.9	147.1 ± 34.9*
	I_to_−I_h_ (pA)	780 ± 189	1817 ± 248*
−70 to +40	I_to_ (pA)	235 ± 84 (10/16)	825 ± 157* (16/16)
	I_to_−I_h_ (pA)	300 ± 82	1557 ± 239*
−60 to +40	I_to_ (pA)	26.7 ± 19 (2/15)	663 ± 164* (15/16)
	I_to_−I_h_ (pA)	50.7 ± 34.8	1412 ± 249*
−50 to +40	I_to_ (pA)	00 ± 0 (0/16)	532 ± 88* (16/16)
	I_to_−I_h_ (pA)	00 ± 0	1163 ± 177*
−40 to +40	I_to_ (pA)	00 ± 0 (0/10)	439 ± 111* (9/9)
	I_to_−I_h_ (pA)	00 ± 0	948 ± 249*
−40 to +80	I_to_ (pA)	182 ± 44 (10/10)	1093 ± 220* (9/9)
	I_to_−I_h_ (pA)	370 ± 52	2176 ± 327*

V_h_ to test step (mV), depolarizing steps from holding potential to voltage indicated; I_to_ (pA), amplitude of I_to_ in pA as the difference between I_to_ peak and the end of 500 msec steps; I_to_−I_h_ (pA), amplitude of the current measured as the difference between I_to_ peak and I_h_; Param, parameters measured; VM cells, data from ventricular myocardial cells; P cells, data from Purkinje cells; τ_f_ (msec) and τ_s_ (msec), fast and slow time constants, respectively, of I_to_ inactivation; Numbers in parenthesis (e.g., 18/18), number of cells in which I_to_ was present over the total number of cells studied; *statistically significant difference between P and VM cells data.

In VM cells, with V_h_ −70 and −60, I_to_ decreased by −40.3% and −93.2%, respectively. With V_h_ −50 and −40 mV there was no apparent I_to_ on depolarization to +40 mV. In P cells, with V_h_ −70, −60, −50, and −40 mV, at +40 mV I_to_ decreased by −9.7%, −27.4%, −41.7%, and −51.9%, respectively. With V_h_ −40 mV, at +80 mV (past the reversal potential of I_Ca_) in VM cells I_to_ was smaller (*) than in P cells. The difference between peak I_to_ and I_h_ (I_to_−I_h_) was larger than I_to_ in both tissues and was much larger (*) in P cells at all V_h_.

Thus, with respect to P cells, in VM cells I_to_ peak: (1) was much smaller; (2) decreased less to a smaller sustained current; (3) was reduced more or altogether eliminated by less negative V_h_; and (4) could be made to reappear with steps from −40 to +80 mV, still being much smaller than in P cells.

### Effect of tetrodotoxin on I_NS_ and of nickel on I_Ca_ component

In P cells, the sodium channel blocker tetrodotoxin (TTX) markedly reduces I_NS_ (Vassalle et al. 2007), including the fraction caused by I_Na3_ (Rota and Vassalle 2003). As in VM cells, sodium currents do not seem to play a dominant role in I_NS_, TTX would not be expected to suppress the NS region. Also, if I_Ca_ is responsible for the inward component positive to I_NS_, the Ca^2+^ channel blocker Ni^2+^ should eliminated it.

**Figure 10:**
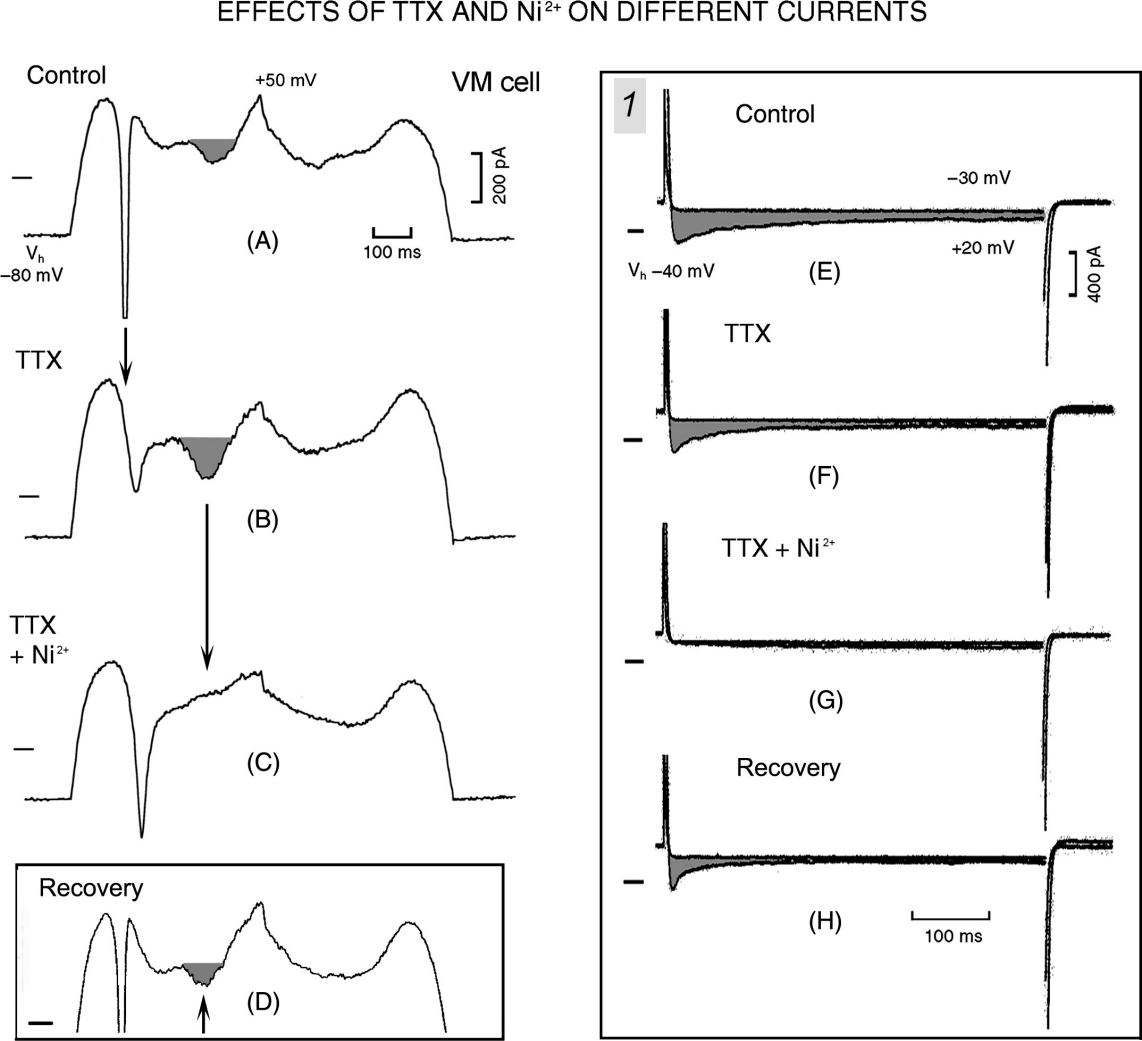
TTX does not eliminate I_NS_ and Ni^2+^ abolishes I_Ca_ component. In A, during the 260 mV sec^−1^ ramp, I_Na1_ was superimposed on I_NS_ which was followed by the I_Ca_ component (shaded area). TTX (30 μmol L^−1^, B) eliminated I_Na1_ (short arrow) but not I_NS_ as confirmed by the presence of I_PS_. Ni^2+^ (2 mmol L^−1^, C) abolished the I_Ca_ component (long arrow). Recovery is shown in D. In inset 1, depolarizing steps from V_h_ −40 mV to +20 mV elicited I_Ca_ (control, E) which was not suppressed by TTX (TTX, F) but was eliminated by Ni^2+^ (TTX + Ni^2+^, G). During the recovery from TTX and Ni^2+^, I_Ca_ returned to control value (recovery, H).

In Figure 10A, control, in the VM cell during the 260 mV sec^−1^ ramp I_Na1_ was superimposed on NS region and the I_Ca_ component was emphasized by shaded area. In Figure 10B, TTX (30 μmol L^−1^) eliminated I_Na1_ (short downward arrow) and only I_NS_ was left. That indeed I_NS_ was not abolished by TTX is confirmed by the fact during the repolarizing ramp a distinct I_PS_ was present as in control.

In the presence of TTX, I_Ca_ (shaded area) was greater than in control. A possible reason for this increase might be that TTX decreases intracellular Na^+^ activity (Abete and Vassalle 1988; Iacono and Vassalle^1990^), thereby increasing the transmembrane Na^+^ gradient. In turn, a larger Na^+^ gradient increases the extrusion of Ca^2+^ through an enhanced Na^+^-Ca^2+^ exchange: this would increase the transmembrane Ca^2+^ gradient and therefore I_Ca_.

In Figure 10C, Ni^2+^ (2 mmol L^−1^) altogether abolished I_Ca_, as emphasized by the long downward arrow. Although 2 mmol L^−1^ Ni^2+^ blocks also the Na^+^-Ca^2+^ exchange, such a block seems unlikely to account for the observed phenomenon, as in the voltage range of I_Ca_, the Na^+^-Ca^2+^ exchange would be operating in the reverse mode generating an outward current.

During the recovery in physiological saline solution (Fig. 10D), I_Na1_ and I_Ca_ reappeared.

In another approach (Fig. 10, inset 1), depolarizing steps were applied from a V_h_ of −40 mV so that Na^+^ channels (but not I_Ca_ channel) were inactivated. In Figure 10E, control, a step from V_h_ −40 mV to −30 mV did not elicit time-dependent currents, whereas depolarization to +20 mV elicited I_Ca_. In Figure 10F, TTX (30 μmol L^−1^) did not suppress I_Ca_ at +20 mV. In Figure 10G, adding Ni^2+^ (2 mmol L^−1^) to TTX solution eliminated I_Ca_ and allowed a small decaying current to appear (presumably an unmasked small I_to_). In Figure 10H, during the recovery from TTX and Ni^2+^, I_Ca_ returned to the control value. Similar results were obtained in *n* = 2.

### Effects of 4-AP and Ba^2+^ on fast and slow ramps in myocardial cells

In Purkinje cells, Cs^+^ and Ba^2+^ (blockers of I_K1_) markedly decreased I_K1_ as well as the slope conductance, but only slightly reduced the outward current at positive potentials (Du and Vassalle 1999). Furthermore, Ba^2+^ eliminated I_K1_ peak but not I_NS_, whereas low V_h_ and TTX eliminated I_NS_ (Rota and Vassalle 2003; Vassalle et al. 2007). As neither a low V_h_ nor TTX abolished I_NS_ in VM cells (present results), in VM cells I_NS_ might be mostly related to Mg^2+^ block of I_K1_. To verify this point, in one experiment fast and slow ramps were applied to test whether Ba^2+^ (by blocking I_K1_ and therefore preventing the block and unblock by Mg^2+^ in the NS and PS regions, respectively) abolished I_NS_ and I_PS_.

**Figure 11:**
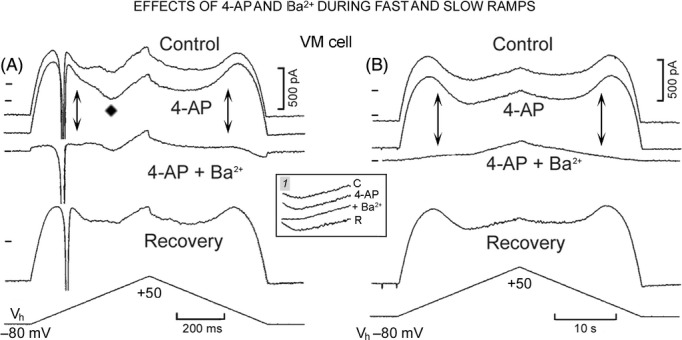
NS and PS regions are abolished by Ba^2+^. In A, 260 mV sec^−1^ ramps were applied in control, in the presence of 4-AP, of 4-AP plus Ba^2+^, and during recovery. In B, the same procedures were applied during 6.5 mV sec^−1^ ramp. The larger I_Ca_ component in the presence of 4-AP is labeled with a rhombus. In the two panels, the left hand double headed arrows emphasize the disappearance of I_NS_ and the right hand double headed arrows that of I_PS_. In inset 1, the current traces recorded between I_Ca_ and ramp peaks in B have been superimposed: C, control; 4-AP, in the presence of 4-aminopyridine; +Ba^2+^, in the presence of 4−AP and Ba^2+^; R, recovery in physiological saline solution.

In Figure 11A, in a VM cell, during a 260 mV sec^−1^ ramp (bottom trace), in control the current exhibited the usual features. In the presence of 4-AP, I_NS_ was little changed whereas I_Ca_ component became larger (+129%, rhombus). While overall the current was less outward, I_K1_ and I_PS_ peaks were somewhat larger. The current between I_Ca_ and ramp peaks was unchanged (+1.6%) as in P cells (Du and Vassalle 1999). In the presence of Ba^2+^, not only the I_K1_ peak, but also I_NS_ and I_PS_ were abolished (as pointed out by double headed arrows). Neither the outward current between I_Ca_ and ramp peaks nor the initial fast decrease in current at beginning of repolarization was suppressed. I_NS_ and I_PS_ reappeared during recovery (bottom current trace).

In Figure 11B, during a 6.5 mV sec^−1^ ramp (bottom trace), in the presence of 4-AP, I_K1_ peak was also somewhat larger and so was I_Ca_. Instead, the current between I_Ca_ and ramp peaks was not affected (−0.6%, see 4-AP trace in Figure 11 inset 1). Ba^2+^ abolished I_K1_ peak and I_NS_, as pointed out by the left hand double headed arrow, leaving only the slowly increasing outward current. As in P cells (Du and Vassalle 1999), the current during the last part of the depolarizing ramp was unaffected (see Ba^2+^ trace in Figure 11 inset 1). During the repolarizing ramp, the current decreased continuously (no I_PS_, as indicated by the right hand double headed arrow). The recovery from the procedures is shown by the bottom current trace and by trace R in Figure 11 inset 1).

Thus, while in P cells Ba^2+^ abolished I_K1_ peak, but not I_NS_ (Rota and Vassalle 2003), in VM cells it abolished I_K1_ peak, I_NS_ and I_PS_. However, neither 4-AP nor Ba^2+^ abolished the increase in outward current between I_Ca_ and ramp peaks.

## Discussion

The present results show numerous dissimilarities between VM and P cell currents. The features involved concern: (1) the slope conductance at the resting potential and its changes during depolarization and repolarization; (2) the sodium currents I_Na1_, I_Na2_, and I_Na3_ (presence or absence, amplitude, threshold, voltage and time dependence, contributions to peak sodium current); (3) outward and inward current during ramps with different slopes; (4) time and voltage dependence of I_K1_ blocking and unblocking; (5) NS and PS regions (voltage range, voltage and time dependence, slope conductance, depolarization vs. repolarization); (6) prevalent role of slowly inactivating sodium currents in the mechanisms underlying NS region in P cells; (7) prevalence of block and unblock of I_K1_ in NS and PS regions, respectively, in VM cells; (8) characteristics of I_Ca_ as well as I_to_ and sustained current; (9) different contributions of I_K1_ and I_to_ to the I-V relation; (10) voltage and time dependence of currents during repolarizing ramps, and (11) response to channels blockers.

We conclude that the differences between VM and P cells involved all the ionic currents studied and account for several electrophysiological differences in resting and action potentials.

### Membrane conductance as a function of voltage and time in VM and P cells

As at negative potentials I_K1_ predominates in determining the I-V relation (Shah et al. 1987), at potentials negative to I_K1_ peak the I-V relation was taken to essentially reflect that of I_K1_. Near the resting potential, the larger slope conductance in VM cells (+100%) is consistent with the findings that I_K1_ in rabbit myocardial ventricular cells is larger than in P cells (Cordeiro et al. 1998). Indeed, the expression of transcripts underlying I_K1_ channel (Kir2.1, Kir2.2, Kir2.3) in human Purkinje fibers is about half that in ventricular myocardium (Gaborit et al. 2007).

The larger slope conductance in VM cells at resting potential could be related to the fact that I_K1_ channels are located also in T-tubules (e.g., Lopatin and Nichols 2001), which are absent in P cells (see Vassalle et al. 1995 for references). In VM cells, the larger I_K1_ peak could possibly be due to the larger resting conductance and/or to lower polyamines level in myocardial cells.

The smaller conductance in P cells would seem at odds with the fact that in 2.7 mmol L^−1^ [K^+^]_o_ their maximum diastolic potential (∼−95 mV; e.g., Vassalle 1965) is more negative than the resting potential in VM cells (∼−80 mV). This discrepancy is due to the presence of the pacemaker current I_Kdd_, (the potassium current underlying diastolic depolarization; Vassalle 1966; Vassalle et al. 1995; Vassalle 2007, 2013), which is present in P cells, but not in VM cells.

Thus, in quiescent P cells in 5.4 mmol L^−1^ [K^+^]_o_ the *resting* potential is ∼−80 mV and does not increase when [K^+^]_o_ is lowered to 2.7 mmol L^−1^ (Vassalle 1965, 1966) due to K^+^-dependent fall in K_1_ channel conductance. However, in cells active in 2.7 mmol L^−1^ [K^+^]_o_, the activation of I_Kdd_ during the AP (Vassalle 1966; Vassalle et al. 1995) is responsible for the voltage undershoot to the maximum diastolic potential. As I_Kdd_ deactivates as a function of time in the diastolic potential range, the pacemaker potential and slope conductance decrease toward the resting potential value (Weidmann 1951; Vassalle 1965, 1966). Therefore, in P cells diastolic conductance at *resting* potential is mostly a function of I_K1_ (as in VM cells) and at the *maximum diastolic potential* of both I_K1_ and I_Kdd_.

During ramp depolarization, the progressive decline of slope conductance to a minimum at I_K1_ peak shows that the gradually smaller increase in outward current is due to I_K1_ inward rectification, rather than a progressive increase of an inward current. At I_K1_ peak, I_K1_ is matched by the inward current, as the outward current stopped increasing and the slope conductance became minimal (Fig. 6). Soon after, in both tissues, the activation of I_Na3_ (together with the decreasing I_K1_) accounts for the initiation of the NS region.

During I_NS_, the reversal and reincrease of pulse current indicate that I_K1_ became smaller than the inward current. In fact, I_K1_ reaches its minimum at the peak of I_NS_ (−20 mV in P cells) as shown by I_K1_ I-V relation (control minus I_K1_ blockers) (Shah et al. 1987; Cordeiro et al. 1998). In P cells, the reversal of pulse current occurred both during the part of I_NS_ due to I_Na3_ (Rota and Vassalle 2003) and that due to the inactivation of I_Na2_ (Bocchi and Vassalle 2008). The relation of the reversed pulse current to the activation and decay of I_Na2_ is demonstrated by the findings that during depolarizing steps the amplitude of pulse current became larger when I_Na2_ appeared, decreased gradually during I_Na2_ slow inactivation, and was markedly reduced by lidocaine (Bocchi and Vassalle 2008).

However, as pulse current reversal in NS range occurred also during the 6.5 mV sec^−1^ ramp (Fig. 6E) when the Na^+^ currents are inactivated (and in VM cells the inactivating I_Na2_ would play little role), the reversal appears to involve also the block of I_K1_ channel. In both cases (increase in Na^+^ currents and decrease in I_K1_) the net current would become inward (I_NS_) and therefore the pulse current would reverse. The role of I_K1_ change in pulse current reversal is supported by the occurrence of the reversal also during I_PS_ (Fig. 6D and E), when any possible residual Na^+^ currents would be inactivated.

### Contribution of I_Na3_, I_Na2,_ and rectification of I_K1_ to NS region in P and VM cells

Our findings show that the mechanisms underlying I_NS_ in P cells differ qualitatively and quantitatively from those in VM cells and account for some previously reported results.

In the presence of TTX and of Ca^2+^ blocker nicardipine in rabbit, the NS region was present in VM cells but not in P cells (Cordeiro et al. 1998). This difference is accounted by the finding that I_Na3_ (Rota and Vassalle 2003) and I_Na2_ (Vassalle et al. 2007; Bocchi and Vassalle 2008) are blocked by TTX. However, when measured as the difference current (control minus Ba^2+^), a small NS region was present also in P cells (Cordeiro et al. 1998), suggesting that, in the absence of Na^+^ and Ca^2+^ currents, the residual I_NS_ was due to a block of I_K1_.

Similarly, in P cells with steps from a V_h_ of −50 mV, the NS region was not always found, but it was present in I_K1_ I-V relation (control minus current in Ba^2+^ or Cs^+^) (Shah et al. 1987). In retrospect, at V_h_ −50 mV the Na^+^ currents would have been inactivated or markedly reduced (Rota and Vassalle 2003; Vassalle et al. 2007; present results). Therefore, the findings of Shah et al. (1987) can also be accounted for by the inactivation of Na^+^ currents at −50 mV and a small contribution of I_K1_ block to I_NS_ in P cells. That in P cells, I_K1_ rectification contributes to I_NS_ is also indicated by a net decrease of radioactive K^+^ efflux in the −60 to −40 mV range (e.g., Vereecke et al. 1980).

In our experiments, in the absence of blockers, with respect to VM cells, in P cells the increase in I_NS_ with the steeper ramps and its decrease or absence with 6.5 mV sec^−1^ ramp are consistent with time-dependent inactivation of I_Na3_ and I_Na2_. In P cells, with V_h_ −80 mV, the smaller I_NS_ in I-V relation of the sustained current is consistent with inactivated I_Na3_ and substantially reduced I_Na2_ by the end of 500 msec steps. In addition, the gradual decrease of I_NS_ with less negative V_h_ in P but not in VM cells (Fig. 4, inset 1) indicates a voltage-dependent inactivation of Na^+^ currents.

Conversely, with 6.5 mV sec^−1^ ramps the much smaller I_NS_ in P cells is explained by I_Na3_ and I_Na2_ being reduced or absent, in agreement with the little effects of TTX on I_NS_ and by the similar values of I_NS_ and I_PS_. Furthermore, with V_h_ −50 mV, during fast ramps the near abolition of I_NS_ in P cells but not in VM cells also points to a greater role of Na^+^ currents.

In P cells, TTX, lidocaine, and low V_h_ markedly reduced I_Na3_ (Rota and Vassalle 2003) as well as I_Na2_ (Vassalle et al. 2007) and so did low [Na^+^]_o_ (Bocchi and Vassalle 2008). Yet, I_NS_ persisted in VM cells with some of these procedures (present results). Furthermore, in P cells, Ba^2+^ abolished I_K1_ peak but not I_NS_ (Rota and Vassalle 2003) whereas it abolished I_K1_ peak, I_NS_, and I_PS_ in VM cells.

These results indicate a predominant role of Na^+^ currents in P cell I_NS_ and of I_K1_ inward rectification in VM cell I_NS_. Indeed, in guinea pig ventricular myocytes, the K^+^ channel opener cromakalim abolished I_NS_ and markedly shortened the action potential (Liu et al. 1990).

As for voltage ranges of Na^+^ currents and of I_NS_, the beginning of I_NS_ during the ramps (∼−58 mV) indicates the participation of I_Na3_ as in P cells this current started at ∼−58 mV (Table 8; Rota and Vassalle 2003) and peaked before or by the end of ramps to −42 mV (Rota and Vassalle 2003). In both P and VM cells, I_Na3_ contributed to I_NS_ prior to I_Na1_, but (its slow inactivation being cut off by I_Na1_) presumably little after I_Na1_.

In P cells, the slowly inactivating I_Na2_ can contribute to I_NS_ after the inactivation of I_Na1_, as I_Na2_ has a −40 mV threshold and is largest at a voltage (−30/−20 mV, Vassalle et al. 2007) which is near to the peak of I_NS_ (−26.5 mV with 260 mV sec^−1^ ramp). I_Na2_ activates also in the absence of I_Na1_ (Fig. 5B and E), in agreement with the findings of Bocchi and Vassalle (2008). Indeed, in those P cells in which I_Na1_ was not present with 260 mV sec^−1^ ramps, I_NS_ peaked at a potential (−29.2 mV, present results) near the I_Na2_ peak.

Therefore, with respect to VM cells, in P cells I_NS_: (1) had a smaller voltage range; (2) was larger with faster ramps; and (3) was smaller with slow ramps, with lower V_h_ and in the presence of TTX. These finding indicate a predominant role of the sodium currents in I_NS_ of P cells and of I_K1_ block in VM cells.

### Dual mechanism of I_K1_ inward rectification and the NS and PS regions in P and VM cells

As for the inward rectification of I_K1_ channel, two mechanisms have been demonstrated: block by intracellular polyamines (channel gating by spermine and spermidine; Lopatin et al. 1994; see Lopatin and Nichols 2001) and block by Mg^2+^ (Matsuda et al. 1987; Vandenberg 1987). The I_K1_ block by spermine and spermidine is time dependent (Ishihara 1997), whereas the block by Mg^2+^ and putrescine is voltage dependent and virtually instantaneous (Ishihara and Ehara 1998).

In guinea pig VM cells, in the absence of internal Mg^2+^, the block by polyamines occurs between −80 and ∼−40 mV whereas, with in the presence of internal Mg^2+^, I_K1_ block is present also between −40 and 0 mV (Ishihara 1997). Furthermore, after a depolarization larger than 0 mV (which would cause Mg^2+^ block), on repolarization to −50 mV there was a sudden transient increase in outward current (see also Shimoni et al. 1992). The amplitude of the outward current was correlated to the degree of Mg^2+^ block during the previous depolarization, indicating that the increase in outward current was due to the removal of Mg^2+^ block. During a repolarizing ramp, the outward current at −50 mV was substantially larger in the presence than in the absence of internal Mg^2+^, indicating the importance of the removal of Mg^2+^ block of I_K1_ for the repolarization of the action potential (Ishihara 1997; Ishihara and Ehara 1998).

Because I_K1_ block by polyamines begins at more negative voltage than that by Mg^2+^ (Ishihara et al. 1989; Ishihara 1997; Ishihara and Ehara 1998), the block of I_K1_ ought to be solely due to polyamines up to I_K1_ peak which with the slowest ramp occurred at −44.2 mV in VM cells (Table 5). Instead, the voltage range of the I_K1_ block by Mg^2+^ (−40 to 0 mV) overlaps the NS region in VM cells.

Time dependence of I_K1_ block by polyamines at the beginning of depolarizing steps would account for the initial decline of the outward current. On step repolarization from positive potentials toward E_K_ the sudden increase in outward current due to instantaneous relief of Mg^2+^ block gradually declined due to time-dependent block by spermine and spermidine (Ishihara 1997). The time constant of the exponential decay of the outward current was ∼5 msec at −50 mV which is close to the τ of ∼6 msec for the current decline on depolarization from V_h_ −80 to ∼−60 mV (Table 4).

As for the symmetrical voltage ranges of NS and PS regions, if the voltage-dependent block of I_K1_ by Mg^2+^ initiates near the beginning of I_NS_, its complete removal at the peak of I_PS_ (*ceteris paribus*) should occur at a similar potential. Indeed, in VM cells, with the slowest ramp, I_NS_ initiated at −44.2 mV and I_PS_ peaked at −44.4 mV and their amplitudes were similar (218 and 197 nA, respectively). The peak of I_NS_ (full block) was at −3.9 mV and the beginning of I_PS_ was 8.9 mV (initiation of block removal) (Table 5).

With faster ramps, other factors modified I_K1_ and I_PS_ peaks. In P cells, the larger I_NS_ with faster ramps implicates additional time-dependent factors such as a greater activation of I_Na3_ and of I_Na2_. In keeping with this conclusion, in P cells with 6.5 mV sec^−1^ ramp, I_NS_ was much smaller and so was I_PS_, as the Na^+^ currents would be inactivated during the depolarizing and repolarizing ramp.

That I_K1_ undergoes a progressively greater inward rectification on depolarization from the V_h_ is also shown by the progressively smaller increase in sustained outward current at the end of 500 msec depolarizing steps (when most of the other currents would be completely or partially inactivated). Actual decline of the sustained current at potentials positive to −50 mV in P and to −40 mV on VM cells reflects the I_NS_ seen during the ramps. The less negative potential at which the sustained current decreased with respect to the beginning of I_NS_ during the ramps might be ascribed to lack of I_Na3_ contribution to the sustained current.

At more negative potentials, block and unblock of I_K1_ might contribute to several changes in the I-V relation during ramps of different steepness. Thus, voltage- and time-dependent block by spermine and spermidine on depolarization from V_h_ −80 mV to I_K1_ peak would account for the gradually smaller increase of outward current during a ramp and for the decreasing slope conductance both in P (Rota and Vassalle 2003; Bocchi and Vassalle 2008; present results) and in VM cells (Fig. 6).

As at less negative potentials the channel block by polyamines is much slower (Ishihara et al. 1989; Ishihara and Ehara 1998), such a block may also contribute to the current changes that occur during the ramps with steeper slopes. A greater lag between faster voltage change and block of I_K1_ channel might be responsible for the increase in magnitude of I_K1_ peak with faster ramps. Also, the larger decrease in outward current during faster repolarizations might include a delay in the removal of I_K1_ block by polyamines.

The fact that during repolarizing 260 mV sec^−1^ ramps, in P cells I_PS_ was much smaller (−81%) than I_NS_ (Table 6) suggests that I_Na3_ and I_Na2_ have a major role in I_NS_ and that the remainder (∼19%) is due to I_K1_ block by Mg^2+^. The removal of I_K1_ block would account for the I_PS_ beginning being more outward (no Na^+^ currents contribution) than the I_NS_ peak and I_PS_ peak being similar to I_NS_ beginning. In contrast, in VM cells the similarity of I_NS_ and I_PS_ (Table 6) suggests that block and unblock of I_K1_ channel by Mg^2+^ were the major mechanisms underlying I_NS_ and I_PS_, respectively.

This is consistent with Mg^2+^ block and unblock being only voltage dependent and with the slowly inactivating I_Na2_ not contributing to either I_NS_ or I_PS_ in VM cells. During the slowest ramps, there would be little or no contribution by Na^+^ currents to either I_NS_ or I_PS_, as supported by the similarity of I_NS_ and I_PS_ in VM as well as in P cells (Table 5).

In VM cells, with 260 mV sec^−1^ ramp the larger difference between I_PS_ peak and I_h_ (Table 6) reflected the almost double I_K1_ upon which I_PS_ was superimposed. In contrast, the similarity of the I_PS_ peak voltage in VM and in P cells is consistent with the voltage dependence of the removal of I_K1_ block by Mg^2+^. With the 6.5 mV sec^−1^ ramp, the much larger I_NS_ and I_PS_ in VM than in P cells support the role of Mg^2+^ block and unblock, respectively, as in both tissues the Na^+^ currents would be largely inactivated during both depolarizing and repolarizing ramps.

Therefore, the time- and voltage-dependent block of I_K1_ by polyamines appears to prevail at potentials negative to the I_K1_ peak whereas the inward rectification of I_K1_ during I_NS_ is attributable to Mg^2+^ block. This conclusion is supported by the findings that in P cells: (1) when I_Na3_ and I_Na2_ were present, I_NS_ was larger than in VM cells and more so the faster the ramp; (2) when I_Na3_ and I_Na2_ were inactivated (repolarizing ramps) I_PS_ was smaller in P cells; and (3) with the slowest ramp (I_Na3_ and I_Na2_ being inactivated during the depolarizing and repolarizing phases), both I_NS_ and I_PS_ were smaller in P cells.

### I_Na3_ in Purkinje and myocardial cells

With the approach of Rota and Vassalle (2003), in P cells I_Na3_ was mostly studied at potentials negative to I_Na1_ threshold. With depolarizing ramps (in the absence of I_Na1_), I_NS_ began at −57.7 mV and peaked before or by the end of the ramp at −42 mV. I_NS_ was attributed to the activation of I_Na3_, as shown by its threshold, the marked reduction by TTX and lidocaine and its little sensitivity of Cs^+^ and Ba^2+^. In contrast to I_Na3_, TTX or lidocaine did not abolish I_CaT_ (Tytgat et al. 1990). In addition, I_Na3_ was markedly reduced by 70 mmol L^−1^ [Na^+^]_o_ and it was not abolished by 100 μmol L^−1^ Ni^2+^ (M. Rota and M. Vassalle, unpubl. experiments). These findings contribute to rule out that I_Na3_ might in actuality be I_CaT_.

Suitably large and slow ramps that did not activate I_Na1_ initiated I_Na3_ at about −60 mV followed at about −40 mV by the activation of I_Na2_ (Rota and Vassalle 2003). In retrospect, these results in P cells separated for the first time the contributions of I_Na3_ and I_Na2_ to I_NS_. That I_Na3_ is a sodium current also in VM cells is shown by its decrease with less negative V_h_ (depolarizing steps, Table 1) or ramps (Table 9).

A population of slowly inactivating Na^+^ channels has been also reported in giant squid action. These channels are much fewer than the normal Na^+^ channels, activate on depolarization to ∼−65 mV whereas I_Na_ threshold is −50 mV, activate maximally at −40 mV and undergo a very slow inactivation (Gilly and Armstrong 1984), similar to I_Na3_. As for the mechanism by which I_Na1_ block I_Na3_ slow inactivation, it appears that fast I_Na1_ inactivation blocks slow inactivation of Na^+^ channels by charge immobilization (Richmond et al. 1998).

As to Na^+^ channel isoforms involved in I_Na3,_ among several Na^+^ channels isoforms cloned (Na_V_1 to Na_V_9; cardiac, neuronal, and skeletal), cardiac Na_V_1.5 isoform has a low TTX-sensitivity, whereas the neuronal (Na_V_1.1, Na_V_1.2, Na_V_1.3, Na_V_1.6) and skeletal muscle (Na_V_1.4) isoforms have a high TTX-sensitivity (Zimmer et al. 2002; Haufe et al. 2005a). The neuronal Na_v_1.2, Na_v_1.3, and Na_v_1.6 isoforms are expressed in VM cells and Na_v_1.1 and Na_v_1.2 (Haufe et al. 2005a) as well as skeletal N_aV_1.4 isoform (Qu et al. 2007) have been identified in P cells. Although I_Na3_ and I_Na2_ are more sensitive to TTX block than I_Na1_, the noncardiac channels isoform involved is not known, as the skeletal muscle Na^+^ channel isoform Na_v_1.4 expressed in P cells is also blocked by low concentrations of TTX (Qu et al. 2007).

The neuronal TTX-sensitive Na^+^ channels were found to contribute to peak sodium current by 22% in P cells and by 10% in VM cells (Haufe et al. 2005b) and therefore to the sodium current responsible for the AP upstroke. Our results show that at the I_Na1_ threshold, I_Na3_ was a sizeable fraction of the total I_Na_ current. However, because I_Na1_ was truncated by the saturation of the amplifier consistently in P cells and often in VM, the I_Na3_ contribution to the total I_Na_ is bound to be overestimated. In addition (as shown by means of double steps), the fast inward component of I_Na2_ would be expected to contribute to the total Na^+^ influx during the upstroke.

I_Na3_ appears to be the link between DD and upstroke of AP in P cells by being responsible (Rota and Vassalle 2003) for the depolarizing phase of ThV_os_ (the oscillatory potentials near the threshold for the upstroke; Vassalle 1965; Spiegler and Vassalle 1995; Berg and Vassalle 2000). Successive ThV_os_ increase progressively in size during diastole until the depolarizing phase becomes large enough to attain the threshold for I_Na1_ (Vassalle 1965; Spiegler and Vassalle 1995; Berg and Vassalle 2000). In P fibers, TTX suppressed the spontaneous discharge by abolishing the upward swing of the late diastolic depolarization, although the fibers could still be electrically driven due to smaller sensitivity of I_Na1_ to TTX (see Fig. 3 by Vassalle and Scidà shown in Vassalle 1980). ThV_os_ are present also in sino-atrial node (Kim et al. 1997) and are essential for both the initiation and maintenance of spontaneous activity of cardiac pacemakers (Vassalle 2007, 2013).

In VM cells, the voltage gap between the resting potential and the threshold for I_Na3_ is closed by the depolarization induced by the conducted AP, as of necessity I_Na3_ threshold is less negative than the resting potential. I_Na3_ would then speed up the attainment of I_Na1_ threshold and the induction of the upstroke.

While a 5 mmol L^−1^ [Na^+^]_o_ has been successfully used to compare Na^+^ currents in normal and diseased VM cells (Pu and Boyden 1997; Valdivia et al. 2005), the low [Na^+^]_o_ solution markedly reduced I_Na1_. This apparently makes it unsuited to study the much smaller I_Na3_, as no slowly inactivating I_Na3_ was apparent in those studies. In our experiments, I_Na3_ was demonstrated under physiological conditions at potentials negative to I_Na1_ threshold. Also, less negative V_h_ markedly reduced I_Na1_ (Table 2) leaving a separately identifiable slowly inactivating I_Na3_ (Table 1).

Thus, the present results show that I_Na3_: (1) was also present in VM cells; (2) had threshold that was negative to that of I_Na1_ and was less negative than that in P cells; (3) inactivated slowly; (4) could occur at potentials less negative than I_Na1_ threshold (as shown by preventing I_Na1_ activation); (5) was less inactivated at a lower V_h_ than I_Na1_; (6) increased in amplitude during depolarizing steps past ∼−60 mV due to its voltage-dependent activation and during faster depolarizing ramps (greater Na^+^ channel availability); (7) contributed to magnitude of total Na^+^ influx associated with I_Na1_; (8) was absent during repolarizing ramps; (9) was associated with a reversal of pulse currents and with an increase in slope conductance during I_NS_; and (10) its slow inactivation was suppressed by I_Na1_.

A limitation of our study is represented by the lack of measurements of membrane capacitance (Cm) in the two cell populations analyzed, posing concerns related to the comparison of ionic current amplitudes. In this regard, values of Cm of 121.9 ± 4.8 pF (Rota and Vassalle 2003) and of 125 ± 6 pF (Han et al. 2000) have been reported for canine P cells, whereas Cm values of 113 ± 6 pF (Han et al. 2000) and of 133.4 ± 6 pF (Pu and Boyden 1997) have been reported for canine VM cells. Thus, the average value of the above capacitances for the P cells is 123.4 pF and that for the VM cells is 123.2 pF.

While P cells have no T tubule system, which is present in VM cells, P cells are larger in size than VM cells. Thus, these two features (cell size and T tubule system) tend to offset each other in determining the surface area in the cell populations.

### The fast sodium current I_Na1_ in P and VM cells

The measurement of I_Na1_ magnitude in P and VM cells was hindered by its being truncated by the saturation of the amplifier. However, the finding that with V_h_ −80 mV I_Na1_ was consistently cut off at −10 nA in P cells but only in 13/18 in VM cells indicates that I_Na1_ was smaller in VM than in P cells. Furthermore, with less negative V_h_ (Na^+^ channels being partially inactivated), I_Na1_ was less often truncated and yet in VM cells I_Na1_ was still smaller than in P cells. Also, the less frequent I_Na1_ activation during the 260 mV sec^−1^ ramp in P cells suggests that during this slower ramp I_Na1_ channels are more susceptible to time-dependent inactivation in P than in VM cells.

Several differences regarding I_Na1_ in P and VM cells have been reported. Thus, total amplitude and maximal rate of rise of AP upstroke were much larger in Purkinje than in ventricular fibers (Baláti et al. 1998). Also, neuronal sodium channels contribute less to the peak sodium current in dog ventricular than in Purkinje fibers (Haufe et al. 2005b). A larger I_Na1_ with a more negative threshold in P cells (present results) would contribute to the faster conduction of P cells with respect to VM cells. Both in nonspontaneous P and VM cells, conducted action potentials would depolarize the membrane to the threshold of I_Na3_, which in turn would allow the attainment of I_Na1_ threshold, as seen during the ramps.

### The slowly inactivating I_Na2_

In VM cells, the absence of a large slowly decaying component of I_Na2_ comparable to that of P cells accounts for the fact that (see Introduction), in contrast to P cells, the AP duration of ventricular myocytes was very little affected by TTX, local anesthetics, veratridine, and high [Na^+^]_o_, suggesting that there is little or no slow decaying I_Na2_ in VM cells under physiological conditions. I_Na2_ is more sensitive to TTX than I_Na1_ (Vassalle et al. 2007) and therefore is possibly due to the activation of neuronal or skeletal muscle isoforms. In P cells, the larger contribution of the noncardiac Na^+^ channels (Haufe et al. 2005b) might be due to both I_Na3_ and I_Na2_. In P cells, the fast activation of I_Na2_ (Bocchi and Vassalle 2008) would contribute to the influx of Na^+^ during the upstroke and its slow inactivation contributes to the longer plateau.

A late I_Na_ was found in canine myocardial cells perfused in a K^+^-free and very low Cl^−^ medium by applying a 2000-msec pulse to −130 mV (to remove steady-state inactivation) prior to depolarizing steps applied at intervals of 30 sec (Zygmunt et al. 2001). Our results show that I_Na2_ is small or absent under physiological conditions in VM cells, in contrast to P cells.

However, the findings of Zygmunt et al. (2001) could suggest that under abnormal situations remodeling may shift the voltage range of the late I_Na_ activation to less negative values. In this regard, it is of interest that I_NaL_ was increased in cardiac failure and yet no differences were found in isoform NaV1.1, 1.3, 1.5 subunits and in the subunit b1 and b2, leading to the conclusion that I_NaL_ increase was not due to a subunit isoform switching or to an altered b subunit expression (Valdivia et al. 2005).

In normal human ventricular myocytes, there is little or no I_Na2_ but in myocytes from patient with hypertrophic cardiomyopathy remodeling leads to the appearance of a late Na^+^ current. Ranolazine, a blocker of the late Na^+^ current, had negligible effect on the action potential duration of normal ventricular myocytes, but shortened the longer AP of the myopathic myocytes and reduced the related arrhythmias (Coppini et al. 2013). Therefore, under some pathological conditions remodeling-related I_Na2_ may induce arrhythmias also in VM cells.

As for role of P cell slowly inactivating I_Na2_ in cardiac arrhythmias, an increase in I_Na2_ (but not of I_Na1_) by neurotoxins anthopleurin or ATX II in Purkinje fibers led to the onset of Torsades de pointes. This electrophysiological mechanism appears responsible for congenital and acquired long QT syndromes (LQTS), as abnormally prolonged repolarizing phase of AP leads to early after-depolarizations (EADs) in Purkinje fibers. In vitro and in vivo, in Purkinje fibers the neurotoxins induced EADs were abolished by concentrations of mexiletene that had little effect on I_Na1_ (El-Sherif and Turitto 2003). The present characterization of differences between P and VM cells add insights relevant to mechanisms underlying some LQTS syndromes, especially the drug-induced acquired syndromes.

### The calcium current in P and VM cells

That I_Ca_ was larger in VM cells was shown by the appearance of a large inward component, which peaked in the +10 to +20 mV range and became more conspicuous after the block of I_to_ by 4-AP. Instead, in P cells, the smaller I_Ca_ appeared as an indentation on I_to_ trace or it was not altogether apparent, even in the presence of I_to_ block by 4-AP.

That the inward component peaking at positive values was due to I_Ca_ was shown by its persistence at low V_h_ and by the abolition by Ni^2+^ of both the inward component during the ramps and I_Ca_ during depolarizing steps. In VM cells, I_Ca_-induced positive shift of the onset of the smaller outward current would contribute to the more positive plateau and the larger I_Ca_ to the greater force of contraction (Lin and Vassalle 1978) with respect to P cells.

As for the identity of Ca^2+^ currents, the L-type (I_CaL_, isoform Ca_v_1.2 containing the pore forming α1C subunit) and T-type (I_CaT,_ isoforms Ca_v_3.1, Ca_v_3.2, and Ca_v_3.3) are expressed both in P and M cells, I_CaT_ being more abundantly expressed in P cells and I_CaL_ predominating in VM cells (see Dun and Boyden 2008). These differences suggest that calcium current recorded in VM cells is of the I_CaL_ type, in accordance with fact that it was recorded with less negative V_h_ and at less negative voltage.

### The transient outward current I_to_ in P and VM cells

With respect to VM cells, in P cells a larger I_to_ contributed to the much larger outward current between I_NS_ and ramp peaks, consistent with the shift of the plateau to more positive values by 4-AP (Kenyon and Gibbons 1979; Dumaine and Cordeiro 2007).

However, our results show that the *total* outward current included different components in P and VM cells, as in VM cells a smaller I_to_ was superimposed on a larger I_K1_. In VM cells, the smaller I_to_ would induce a smaller phase 1 repolarization and (together with the larger I_Ca_) would contribute to the more positive plateau than in P cells (e.g., see Fig. 1 in Baláti et al. 1998).

The finding that 4-AP abolished the peak I_to_, but only reduced the sustained current at the end of depolarizing steps and the outward current during latter part of depolarizing ramps agrees with the results shown in other reports (no elimination of sustained current by 4-AP; Kenyon and Gibbons 1979; Cordeiro et al. 1998; Han et al. 2000). Therefore, I_to_ may have a time-independent background component or it may not inactivate completely (Kenyon and Gibbons 1979).

Also, there might be the contribution of an unidentified outward current present at positive potentials which is not eliminated by 0 mmol L^−1^ [K^+^]_o_ (Ishihara et al. 1989). Even I_K1_ block by Cs^+^ and by Ba^2+^ did not suppress the increase in outward current at potentials positive to −40 mV, as shown by figures in Shah et al. (1987) and reported by Du and Vassalle (1999). This is consistent with the finding that ^42^K efflux at potential positive to −30 mV was not blocked by Cs^+^ (Vereecke et al. 1980).

This could be due either to the voltage dependence of Cs^+^ induced block as it is for Ba^2+^ (Hirano and Hiraoka 1986) or to a Cs^+^ insensitive outward rectifier. In the experiments of Cordeiro et al. (1998), 0.1 mmol L^−1^ Ba^2+^ reduced the inward I_K1_, but did not decrease the large outward current at positive potentials. In P cells (Du and Vassalle 1999) and in VM cells, 2 mmol L^−1^ Ba^2+^ eliminated I_K1_ peak, but did not eliminate the outward current at positive potentials.

However, this *per se* does not rule out a contribution of I_K1_ to the outward current, as Ba^2+^ block of I_K1_ is removed on depolarization and slowly reestablished on repolarization (Hirano and Hiraoka 1986; Imoto et al. 1987; Valenzuela and Vassalle 1991). Therefore, in P cells after I_NS_ peak, the increase in outward current includes I_to_ (which is not blocked by Ba^2+^, e.g., Cordeiro et al. 1998; Du and Vassalle 1999) and a current which is not suppressed by 4-AP, Ba^2+^, Cs^+^, or 0 mmol L^−1^ [K^+^]_o_. The delayed rectifier I_K_ could contribute to the outward current, but is blocked by Ba^2+^, albeit to a lesser extent at positive values (Osterrieder et al. 1982). In addition, in P cells I_K_ is rather small (<20 pA, Cordeiro et al. 1998) or undetectable (Kenyon and Gibbons 1979). Indeed, in the presence of 4-AP the outward current did not increase with depolarizing steps from V_h_ −40 mV (Fig. 9).

The increase in peak outward current with steeper ramps in P cells could be due to a larger I_to_ and/or to a lag in I_K1_ block by polyamines which is much slower at positive potentials (Ishihara et al. 1989). In VM cells, with steeper ramps the failure of the ramp peak current to appreciably increase suggests little change in I_to_ whereas the increase in current between ramp peak and I_h_ indicates a role of I_K1_.

On repolarization, with respect to VM cells, in P cells the greater fall in current is bound to reflect the much greater I_to_ activated on depolarization, as 4-AP reduced both I_to_ and the decrease in current on repolarization. The finding that on repolarization the decrease in outward current reached a more negative peak in P cells (∼−16 mV) than in VM cells (∼+7 mV) suggests that on depolarization the less negative reincrease of the outward current in M cells does not entirely depend on being masked by a larger I_Ca_.

In P cell, I_to_ induces the large phase 1 repolarization of AP, and keeps the plateau at more negative level (Kenyon and Gibbons 1979). In rabbit P cells, 4-AP slowed phase 1 repolarization and shifted the plateau to positive potentials, while having in VM cells a much smaller effect on phase 1 repolarization and no effect on the plateau (Cordeiro et al. 1998).

In P cells, the persistence of the bulge current during the slow ramps and its elimination by 4-AP (which only reduced the sustained current) suggest that in P cells the bulge current is related to the activation of I_to_ whereas the 4-AP resistant increase in outward current toward the ramp peak might reflect a voltage-dependent increase in the sustained current. Another difference is that in VM cells I_to_ markedly decrease with lower V_h_ whereas in P cells a smaller I_to_ persisted even with V_h_ −40 (Table 11), consistent with the persistence of the bulge current with V_h_ −50 mV (Du and Vassalle 1999).

The greater I_to_ in canine P cells has been also found rabbit P cells versus VM cells (Cordeiro et al. 1998). However, in P cells the larger I_to_ and the smaller I_K1_ contributions to the total outward current at the plateau may be important in different situations. For example, an increase rate of discharge (tachycardia) may modify I_to_ (due to its slow recovery; e.g., Cordeiro et al. 1998) more than I_K1_.

In agreement with the larger I_to_ in P cells, Kv3.4 is more abundant at both the mRNA and protein level in Purkinje fibers than in ventricular myocardium. As the Kv3.4 subunit carries a TEA-sensitive I_to_ outward current, Kv3.4 current may be responsible for the large TEA-sensitive component of I_to_ in canine Purkinje cells (see Schram et al. 2002 for review). Among the differences between I_to_ in P and in VM cells is the different sensitivity to various blockers (e.g., TEA) and a smaller time constant of inactivation in VM cells (Han et al. 2000).

Thus, with respect to the P cells, in VM cells I_to_: (1) became apparent at more positive potentials during the ramps; (2) did not undergo an enhancement (the “bulge”) during depolarizing ramps; (3) was smaller as measured from I_to_ peak either to the end of the step or with respect to I_h_; (4) was abolished by 4-AP with a reduced sustained current (as in P cells); (5) had a smaller time constant of inactivation (in agreement with the finding of Han et al. 2000); (6) was associated with smaller slope conductance; (7) during repolarizing ramps, was smaller and had a smaller voltage range; and (8) was inactivated by lower V_h_.

## General conclusions

The present results indicate substantial differences between P and VM cells that bear on the different functions of P (conduction and pacemaker activity) and VM cells (contraction). In both tissues, I_K1_ is important for a normal resting potential and its inward rectification is important in more than one way. In P cells, the smaller I_K1_ conductance at the resting potential is essential for pacemaker function in two respects: (1) it keeps the resting potential less negative than E_K_ and therefore it allows the undershoot to the maximum diastolic potential by the pacemaker current I_Kdd_; and (2) I_K1_ inward rectification by polyamines contributes to diastolic depolarization caused by time-dependent decay of I_Kdd_ and therefore to the attainment of threshold for I_Na3_. I_Na3_ initiates I_NS_ and the associated depolarization allows the attainment of I_Na1_ threshold.

Especially in VM cells, I_K1_ inward rectification caused by Mg^2+^ block in the voltage range −40 to 0 mV facilitates the attainment of threshold for Na^+^ currents and contributes (see Shimoni et al. 1992) to maintain the plateau. The large and fast I_Na1_ cuts short the no longer needed I_Na3_ and (being large) rapidly depolarizes the membrane and causes the overshoot to positive potentials (fast and large depolarization for rapid conduction). The depolarization induced by I_Na1_ allows the attainment of the thresholds for other currents in an orderly fashion. I_Na2_ (by inactivating slowly in P cells) contributes to their long plateau, thereby preventing the reentry of excitation from myocardium. Once the thresholds for I_to_ and I_Ca_ have been reached, at the end of the upstroke I_Na1_ is rapidly inactivated (thereby preventing a useless Na^+^ influx).

The fast activation of I_to_ eliminates the overshoot which is no longer needed for fast conduction. In the meanwhile, I_Ca_ initiates the events leading to contraction and its inactivation balances the inactivating I_to_ at the plateau over a background of I_K1_ inward rectification. In VM cells, I_Ca_ shifted the outward current reincrease by 35 mV and therefore would contribute to the more positive plateau. In P cells, the inwardly rectifying I_Kdd_ becomes activated and whatever slowly decaying I_Na2_ is not inactivated by the end of the plateau becomes deactivated as a function of voltage (Bocchi and Vassalle 2008). During the repolarization, the removal of Mg^2+^ block of the K_1_ channels leads to I_PS_ and speeds up phase 3 repolarization, which, in turn, removes the polyamines block. In P cells, I_Kdd_ begins to increase at about I_PS_ peak (∼−50 mV, Vassalle et al. 1995) leading to undershoot of resting potential, followed diastolic depolarization.

Among the differences between the two tissues, in P cells the more negative thresholds for the Na^+^ currents, the larger I_Na1_ and the larger overshoot contribute to earlier activation and faster conduction. The larger I_to_ contributes to less negative plateau and the slowly inactivating I_Na2_ to the longer AP. The larger sodium influx may involve a higher Na^+^-K^+^ pump activity, which is the major mechanisms underlying overdrive suppression (Vassalle 1977). Thus, the outward current created by Na^+^-K^+^ pump activity maintains the diastolic depolarization negative to I_Na3_ threshold, so that P cell spontaneous discharge is suppressed when not needed during sino-atrial rhythm.

In VM cells, the larger resting conductance contributes to set the resting potential near the K^+^ equilibrium potential, as a resting potential lower than E_K_ is needed only for pacemaker activity (see Vassalle 2007, 2013). In VM cells, the smaller I_Na1_ is consistent with the smaller rate of rise of the smaller upstroke (e.g., Baláti et al. 1998), as the conduction path is shorter (from Purkinje network to epicardium). In VM cells, the larger I_Ca_ contributes to stronger contraction (Lin and Vassalle 1978); the smaller I_to_ together with the positive range of the larger I_Ca_ contributes to the more positive plateau. The lack of slowly inactivating I_Na2_ contributes to a shorter AP (which in turn regulates the duration of twitch and of diastole) and the greater I_K1_ and removal of its block during the larger I_PS_ contributes to an earlier and faster phase 3 repolarization.

The definition of these differences is a precondition also for the understanding of deranged function under pathological conditions.

## Author Contributions

The experiments were carried out in Dr. Vassalle's lab in the Department of Physiology at SUNY, Downstate Medical Center, Brooklyn, NY. Dr. Vassalle conceived and designed the experiments, participated in part of the experiments, analyzed the data, drew the conclusions and wrote the manuscript. Dr. Bocchi participated in the collection, analysis, and interpretation of the data, their statistical evaluation, and supported various aspects of the writing of the manuscript. All authors approved the final version of the manuscript and qualify for authorship, and all those who qualify for authorship are listed.
